# Sympathetic regulation of bone homeostasis and remodeling: molecular mechanisms, pathophysiological roles, and therapeutic implications

**DOI:** 10.3389/fimmu.2026.1908275

**Published:** 2026-08-24

**Authors:** Changshun Huang, Shouxiang Kuang, Xi Chen, Shengwen Fu, Guodong Wang, Yang Li, Hongdong Tan, Chenggui Zhang

**Affiliations:** 1 Department of Orthopaedics, Shandong Provincial Hospital Affiliated to Shandong First Medical University, Jinan, Shandong, China; 2 School of Clinical and Basic Medicine, Shandong First Medical University and Shandong Academy of Medical Sciences, Jinan, Shandong, China; 3 Department of Spinal Infection Surgery, Shandong Public Health Clinical Center, Shandong University, Jinan, Shandong, China

**Keywords:** bone metabolism, bone-related disorders, osteoimmunology, skeletal homeostasis, sympathetic nervous system

## Abstract

Bone metabolic disorders, such as osteoporosis, osteoarthritis, and rheumatoid arthritis, are characterized by disrupted skeletal homeostasis and represent major challenges in musculoskeletal health. Bone remodeling has long been interpreted as a coordinated process between osteoblast-mediated bone formation and osteoclast-mediated bone resorption. However, emerging evidence indicates that bone is not merely a structural tissue, but a dynamic regulatory organ influenced by neural, endocrine, immune, metabolic, and vascular signals. Among these regulatory systems, the sympathetic nervous system (SNS) has attracted increasing attention as an important efferent pathway linking central physiological states with local skeletal remodeling. Sympathetic signals reach skeletal tissues through organized peripheral innervation and regulate bone-related cells through multiple pathways involving neurotransmitters, neuropeptides, receptors, and regulatory factors. Beyond direct regulation of osteoblasts, osteoclasts, osteocytes, chondrocytes, and skeletal progenitors, SNS-related pathways also shape the osteoimmune microenvironment by modulating immune-cell recruitment, macrophage polarization, cytokine production, inflammasome activation, and immune-related osteoclastogenesis. Dysregulation of these pathways contributes to bone-related disorders, including osteoporosis, osteoarthritis, rheumatoid arthritis-related bone destruction, intervertebral disc degeneration, and impaired fracture healing. By integrating anatomical, molecular, osteoimmune, and disease-related evidence, this review provides a comprehensive framework for understanding sympathetic regulation in bone metabolism and may support the development of more precise neuroregulatory strategies for skeletal diseases.

## Introduction

Bone metabolic disorders, including osteoporosis, osteoarthritis, rheumatoid arthritis-related bone destruction, intervertebral disc degeneration, and impaired fracture healing, are major manifestations of disrupted skeletal homeostasis (1–5). Although these diseases differ in anatomical location and clinical presentation, they commonly involve abnormal bone formation, excessive bone resorption, cartilage degeneration, inflammatory activation, or defective tissue repair. With population aging and the increasing burden of chronic musculoskeletal diseases, clarifying the regulatory mechanisms that maintain skeletal homeostasis is essential for understanding disease pathogenesis and identifying new therapeutic targets.

Bone remodeling has traditionally been explained by the coordinated coupling between osteoblast-mediated bone formation and osteoclast-mediated bone resorption (6, 7). However, skeletal homeostasis is not determined by bone cells alone. Increasing evidence indicates that bone remodeling and repair are regulated by a complex local and systemic network involving neural, endocrine, immune, metabolic, and vascular signals (8–21). This expanded view has shifted attention from local bone cell coupling to higher-order regulatory systems that coordinate skeletal responses at the organismal level.

Among these systems, the nervous system has emerged as an important modulator of bone metabolism and skeletal homeostasis. Peripheral nerves are widely distributed in the periosteum, cortical bone canals, bone marrow, epiphyseal regions, and osteochondral junction, providing the anatomical basis for neuro-skeletal communication (22). Upon stimulation, skeletal nerve fibers release neurotransmitters, neuropeptides, neurotrophins, and axon guidance-related factors that regulate bone cells and other cellular components of the skeletal microenvironment (23–26). These findings indicate that neural regulation represents an essential route by which systemic physiological signals are transmitted to local skeletal tissues.

Within this framework, the sympathetic nervous system has received particular attention as a major efferent branch of the autonomic nervous system. In addition to mediating classical stress responses, sympathetic signaling participates in endocrine regulation, energy metabolism, vascular control, immune modulation, and tissue repair, thereby linking central physiological states with peripheral bone metabolism (27). Norepinephrine-mediated adrenergic signaling, especially through β-adrenergic receptors, has been implicated in suppressing osteoblast activity, promoting osteoclastogenesis, and regulating RANKL-dependent bone resorption (28–31). In addition to the direct actions of sympathetic neurotransmitters and co-transmitters, including norepinephrine and neuropeptide Y, sympathetic regulation of bone metabolism is influenced by several upstream and local modulatory systems. These include leptin-dependent central sympathetic activation, serotonin-related regulation of sympathetic tone, endocannabinoid-mediated modulation of norepinephrine release, and receptor-mediated dopaminergic effects on skeletal cells (8, 32). Importantly, sympathetic signaling also regulates the osteoimmune microenvironment by influencing inflammatory cytokine production, macrophage polarization, synovial inflammation, and marrow immune niches, thereby indirectly affecting osteoblast and osteoclast activity (31, 33, 34). Although recent reviews have summarized the major mechanisms and therapeutic implications of sympathetic regulation in bone-related disorders, several important advances have emerged from recent original studies. These include sympathetic regulation of bone marrow immune niches, interactions among immune cells, vascular cells, and mesenchymal cells during skeletal repair, neutrophil-specific adrenergic signaling in fracture healing, endothelial senescence-associated bone loss, and locally protective sympathetic effects in inflammatory joint environments. Building on the existing literature, the present review organizes current evidence into direct skeletal regulation and indirect osteoimmune regulation. It also emphasizes the context-dependent nature of sympathetic signaling and provides an integrated description linking the anatomical distribution of sympathetic innervation with local inflammatory conditions, target-cell responses, disease-specific effects, and tissue repair outcomes. In addition, we summarize upstream and local modulatory pathways, including the norepinephrine transporter, leptin, serotonin, endocannabinoid, dopaminergic, and cholinergic/VIPergic signaling. We also incorporate circadian coupling as a temporal dimension of sympathetic regulation, highlighting how central and peripheral clocks interact with adrenergic signaling to influence osteoblast activity, osteoclastogenesis, bone-marrow niche function, and skeletal remodeling.

**Figure 1:**
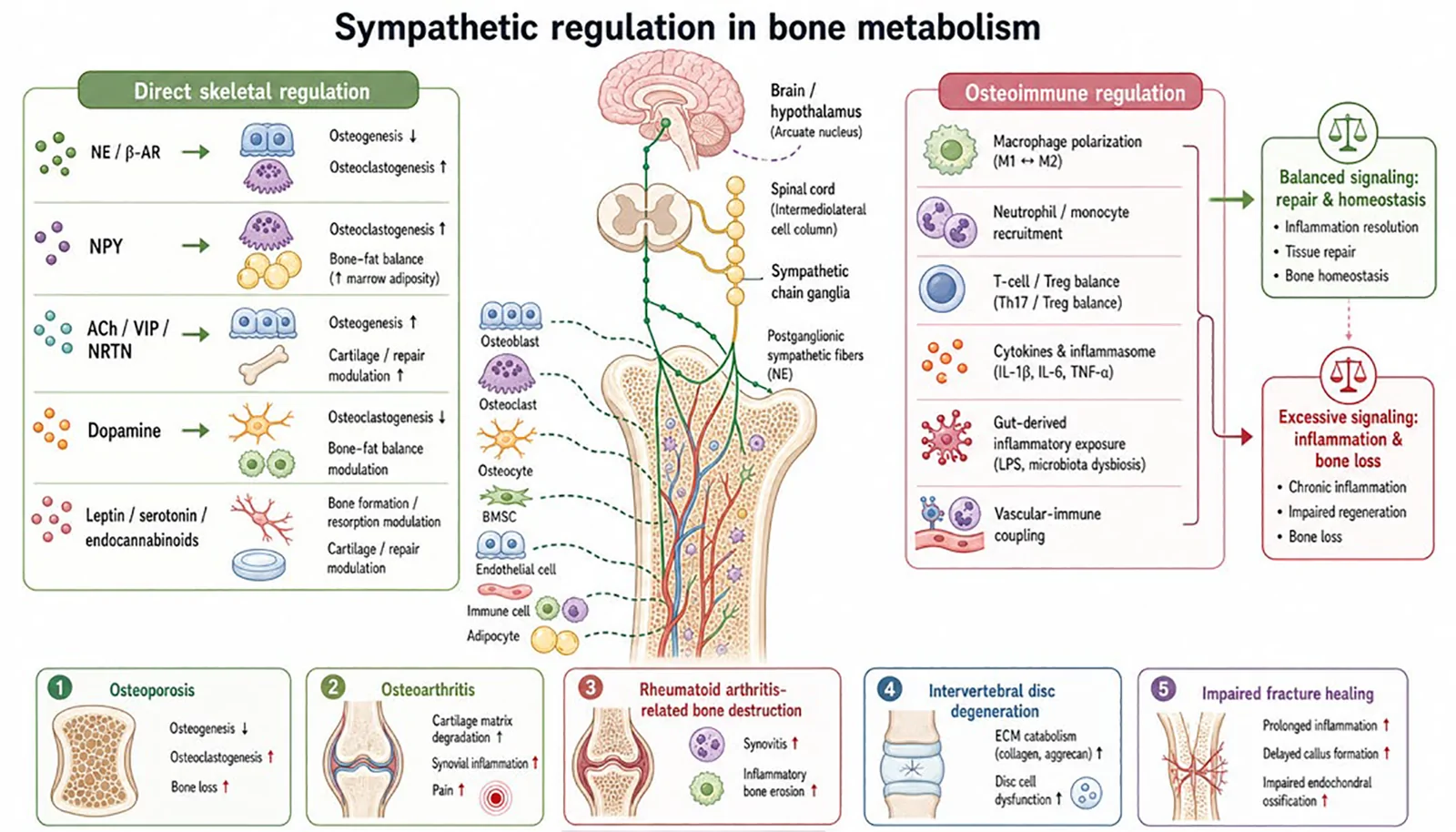
Sympathetic regulation in bone metabolism. Sympathetic regulation of skeletal remodeling involves both direct neuro-skeletal signaling and indirect osteoimmune modulation. Central sympathetic output reaches the bone microenvironment through sympathetic ganglia and postganglionic fibers, establishing functional communication between the nervous system and skeletal tissues. Sympathetic neurotransmitters and neuromodulators, including norepinephrine (NE), neuropeptide Y (NPY), acetylcholine (ACh), vasoactive intestinal peptide (VIP), dopamine, leptin, serotonin, endocannabinoids, and neurturin (NRTN), act on osteoblast-lineage cells, osteoclasts, osteocytes, bone marrow stromal cells, endothelial cells, immune cells, and adipocytes. Direct neuro-skeletal signaling regulates osteogenesis, osteoclastogenesis, osteocyte-mediated remodeling, marrow adipogenesis, angiogenesis, cartilage homeostasis, and bone repair. Osteoimmune modulation further involves macrophage polarization, neutrophil and monocyte recruitment, T-cell responses, cytokine production, inflammasome activation, vascular–immune coupling, and gut–bone inflammatory communication. Dysregulated sympathetic activity contributes to osteoporosis, osteoarthritis, rheumatoid arthritis-associated bone destruction, intervertebral disc degeneration, and impaired fracture healing.

Accordingly, this review integrates anatomical, molecular, osteoimmune, temporal, and disease-related evidence to provide a more complete understanding of sympathetic regulation in skeletal homeostasis and remodeling (**Figure 1**). By linking sympathetic innervation with cellular responses, inflammatory conditions, disease progression, and tissue repair, this review further clarifies the context-dependent effects of sympathetic signaling and its potential therapeutic implications in bone-related disorders.

## Anatomical basis of sympathetic innervation in skeletal tissues

### Peripheral autonomic routes to skeletal tissues

The peripheral nervous system provides the anatomical route through which neural signals from the central nervous system reach skeletal tissues. Within the peripheral nervous system, neuronal cell bodies are located in sensory and autonomic ganglia, and their axons extend toward peripheral target tissues (35, 36). In the skeleton, sensory and autonomic nerves coexist but differ in anatomical origin and functional orientation (37). Sensory neurons innervating the limbs mainly originate from dorsal root ganglia and transmit afferent signals related to pain, mechanical stimulation, and tissue injury, whereas autonomic nerves form efferent pathways that regulate peripheral organs, including bone (38).

Among autonomic inputs to the appendicular skeleton, sympathetic innervation is predominant. Preganglionic sympathetic neurons arise from the thoracolumbar spinal cord and project through the ventral spinal roots to synapse with postganglionic neurons in the paravertebral sympathetic chain or prevertebral ganglia (39). From these ganglia, postganglionic sympathetic fibers extend toward skeletal tissues, forming a direct anatomical connection between central autonomic output and the bone microenvironment.

### Spatial distribution of sympathetic fibers within bone and joint tissues

Sympathetic fibers within bone show specific spatial distribution patterns rather than a uniform arrangement (39, 40). They are predominantly localized in the periosteum and bone marrow, and are also present within nutrient canals that traverse the mineralized bone matrix (41, 42). These regions are functionally important skeletal microenvironments. The periosteum participates in appositional growth, cortical remodeling, and bone repair, whereas the bone marrow supports hematopoiesis, remodeling, and skeletal progenitor activity (43). Thus, the distribution of sympathetic fibers in the periosteum and bone marrow suggests their involvement in local skeletal remodeling and marrow niche regulation.

Within long bones, this spatial organization is further compartmentalized. Quantitative analyses indicate that tyrosine hydroxylase (TH)-positive sympathetic fibers are enriched in the metaphysis and diaphysis but are relatively sparse in the epiphysis (44). In joint-associated tissues, TH-positive sympathetic fibers have been identified in the synovial membrane, subchondral bone, and periosteum, whereas normal articular cartilage is generally considered to lack sympathetic innervation (29, 39, 45). This distribution suggests that sympathetic nerves may participate in joint remodeling and degeneration primarily through innervated periarticular tissues rather than through direct innervation of normal cartilage.

In addition to their regional distribution, sympathetic fibers within bone also exhibit a defined developmental trajectory. In murine long bones, autonomic innervation develops postnatally. NPY-positive fibers can be detected in the femoral and tibial marrow cavity at postnatal day 6, and fibers co-expressing NPY and TH become more evident in epiphyseal regions and periarticular cartilage membranes by postnatal day 10 (44). These findings indicate that sympathetic innervation is not a fixed anatomical feature, but a developing network with both spatial and temporal organization.

### Neurochemical phenotypes of skeletal sympathetic fibers

Beyond their spatial and temporal organization, bone-innervating sympathetic fibers can also be distinguished by their predominant neurotransmitter phenotype. Classical sympathetic postganglionic fibers are noradrenergic (46). In skeletal tissues, these fibers are commonly identified by TH expression, which reflects their capacity for catecholamine synthesis (47). TH-positive sympathetic fibers therefore represent an important local source of norepinephrine within the bone microenvironment (39, 48), providing the anatomical basis for the norepinephrine–adrenergic receptor pathway discussed in later sections (49).

However, skeletal sympathetic innervation should not be regarded as a purely catecholaminergic system. Cholinergic nerve fibers associated with acetylcholine and vasoactive intestinal peptide have also been identified in skeletal tissues, indicating that bone-innervating autonomic fibers include non-noradrenergic phenotypes (45, 50). The origin of skeletal cholinergic innervation remains incompletely resolved. Earlier retrograde tracing studies suggested a parasympathetic contribution (51), whereas more recent evidence indicates that sacral autonomic outflow may be sympathetic in nature (52). In addition, neonatal sympathetic fiber ablation reduces both adrenergic and cholinergic fibers in bone, supporting the possibility that at least a subset of skeletal cholinergic fibers is sympathetic-associated (53).

This neurotransmitter-based distinction provides an anatomical basis for the functional heterogeneity of sympathetic regulation in bone. Noradrenergic fibers mainly support the classical norepinephrine-related pathway, whereas cholinergic-associated fibers may participate in marrow niche regulation, hematopoietic cell trafficking, hematopoietic stem cell quiescence, and bone formation. Thus, skeletal sympathetic innervation should be understood as a heterogeneous autonomic network rather than a uniform noradrenergic input.

### Site-specific patterns of sympathetic innervation in the skeleton

The organization of skeletal innervation also differs across anatomical sites. Long bones and craniofacial bones are innervated by nerves with different origins and anatomical routes (54, 55). In the limbs, sensory nerves mainly arise from dorsal root ganglia, and autonomic innervation is predominantly sympathetic. In contrast, innervation of the craniofacial skeleton is associated with cranial nerve ganglia. Calvarial bone is surrounded by intracranial dura and extracranial periosteal sheaths, both of which contain sensory, sympathetic, and parasympathetic nerve endings (35, 56).

This anatomical arrangement gives the calvaria an innervation pattern distinct from that of long bones. In mature calvaria, sutures are considered major passageways for nerve fibers between the dura and periosteum. Available evidence suggests that these sutures are dominated by sensory nerves, with only a small proportion of sympathetic nerves (57). This pattern differs from the sympathetic enrichment observed in the periosteum, marrow, synovium, and subchondral bone of long bones and joint-associated tissues.

**Figure 2:**
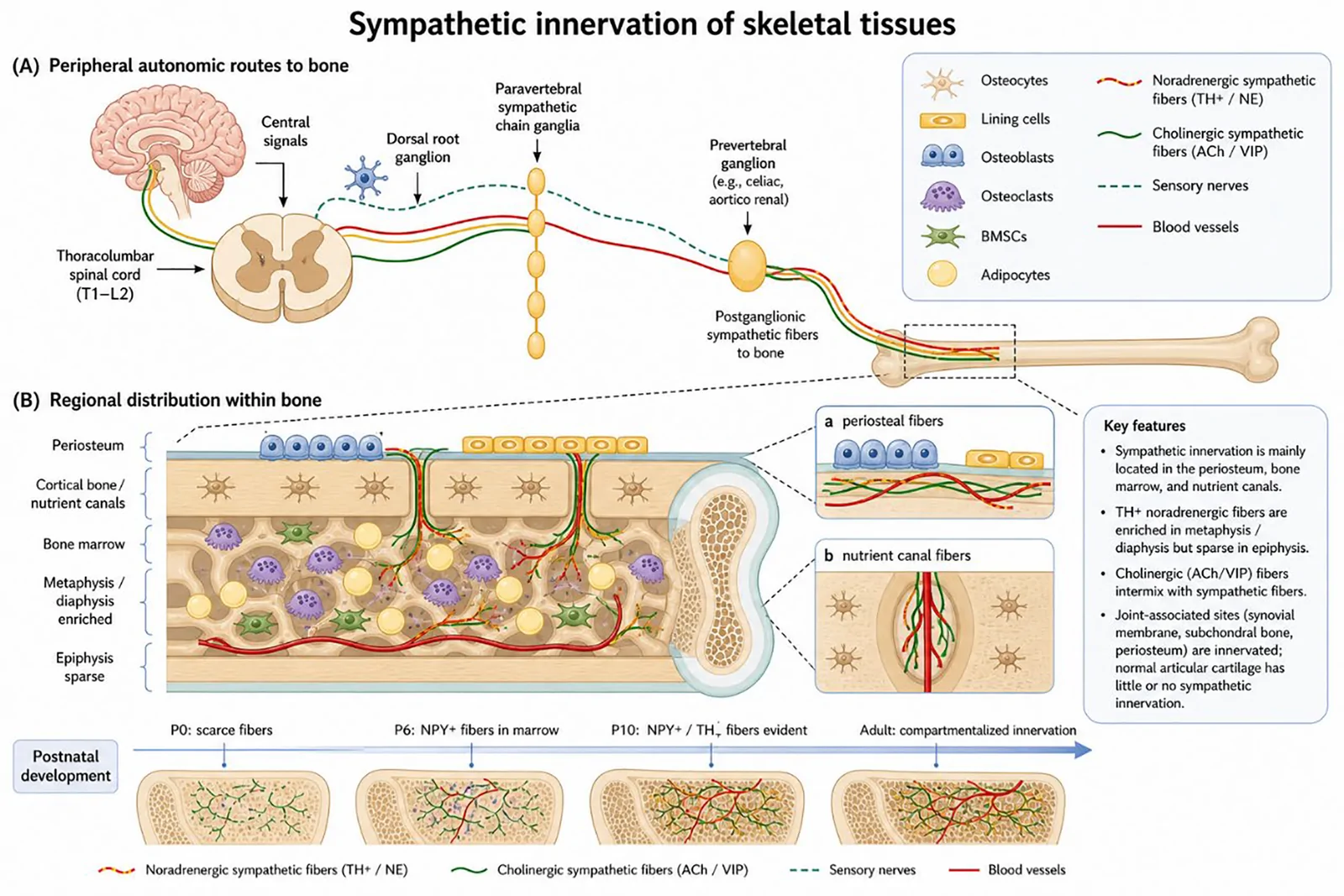
Sympathetic innervation of skeletal tissues. Sympathetic innervation of bone follows defined peripheral autonomic routes and displays marked regional and developmental heterogeneity. **(A)** Central autonomic signals are relayed through the thoracolumbar spinal cord, sympathetic chain ganglia, and prevertebral ganglia before reaching bone via postganglionic sympathetic fibers. Noradrenergic fibers, cholinergic sympathetic-associated fibers, sensory nerves, and blood vessels enter skeletal tissues through periosteal surfaces, nutrient canals, and marrow-associated vascular routes. **(B)** Within bone, sympathetic fibers are enriched in the periosteum, bone marrow, nutrient canals, metaphysis, and diaphysis, but are relatively sparse in the epiphysis. These fibers are closely associated with osteocytes, bone-lining cells, osteoblasts, osteoclasts, bone marrow stromal cells, adipocytes, and vascular structures, forming a neurovascular niche for skeletal remodeling and repair. During postnatal development, skeletal innervation progresses from sparse early nerve fibers to NPY-positive and TH-positive marrow fibers and compartmentalized adult innervation. This spatiotemporal organization provides the anatomical basis for sympathetic regulation of bone homeostasis and disease-associated remodeling.

Taken together, sympathetic innervation of skeletal tissues is organized across several anatomical levels, including peripheral autonomic routes, regional distribution within bone, developmental timing, neurotransmitter phenotype, and skeletal site. This anatomical complexity should be considered when interpreting the role of sympathetic nerves in bone remodeling, cranial repair, joint degeneration, and skeletal disease progression, and may partly explain why sympathetic manipulation produces context-dependent skeletal effects across different anatomical regions and disease models (**Figure 2**).

## Direct sympathetic regulation of bone metabolism: molecular and cellular mechanisms

The anatomical organization of sympathetic fibers in skeletal tissues provides the structural basis for their direct regulation of bone metabolism (58). As described above, sympathetic fibers are distributed in metabolically active skeletal compartments, including the periosteum, bone marrow, nutrient canals, synovium, and subchondral bone. These locations place sympathetic nerves in close proximity to osteoblast-lineage cells, osteoclast precursors, osteocytes, mesenchymal stem/stromal cells, chondrocytes, marrow adipocytes, and vascular-associated stromal components. Therefore, sympathetic signaling is anatomically positioned to influence bone formation, bone resorption, cartilage matrix metabolism, marrow niche activity, and skeletal repair.

**Figure 3:**
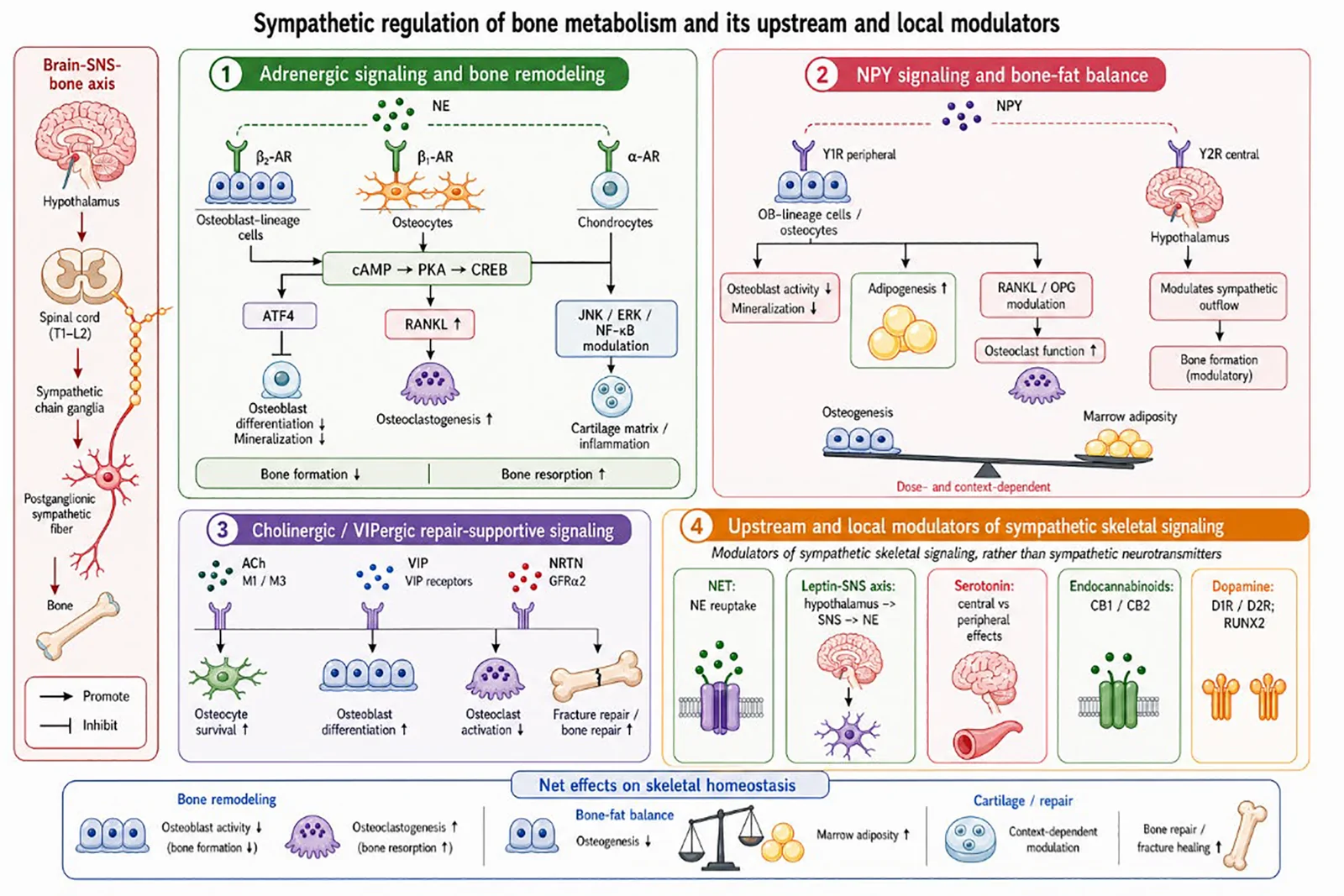
Sympathetic regulation of bone metabolism and its upstream and local modulators. Direct sympathetic regulation of bone metabolism involves adrenergic, NPY-related, cholinergic/VIPergic, and upstream or local modulatory mechanisms. Sympathetic signals from the brain–SNS–bone axis reach skeletal tissues through postganglionic fibers and act on osteoblast-lineage cells, osteocytes, chondrocytes, osteoclasts, marrow adipocytes, and local regulatory niches. (1) Adrenergic signaling, mainly mediated by norepinephrine (NE) and β-adrenergic receptors (β-ARs), regulates osteoblast activity, osteoclastogenesis, osteocyte signaling, and cartilage matrix homeostasis. (2) Neuropeptide Y (NPY) signaling modulates bone remodeling and marrow adiposity through peripheral Y1 receptor-dependent skeletal effects and central Y2 receptor-dependent regulation of sympathetic outflow. (3) Cholinergic and VIPergic sympathetic-associated signaling, involving acetylcholine (ACh), vasoactive intestinal peptide (VIP), and neurturin (NRTN), contributes to osteocyte survival, osteoblast differentiation, osteoclast regulation, and fracture repair. (4) The norepinephrine transporter (NET), leptin, serotonin, endocannabinoid signaling, and dopaminergic signaling act as upstream or local modulators of sympathetic skeletal regulation rather than as equivalent neurotransmitters released from bone-innervating sympathetic fibers. NET regulates local NE reuptake, leptin influences hypothalamic sympathetic outflow, serotonin exerts distinct central and peripheral effects, and endocannabinoid and dopaminergic pathways modify skeletal responses through receptor-dependent mechanisms. Together, these pathways produce context-dependent effects on bone remodeling, marrow adiposity, cartilage homeostasis, and skeletal repair.

Direct sympathetic regulation of bone metabolism is mediated by multiple neurotransmitter-, neuropeptide-, and receptor-dependent pathways (58–60). Among them, norepinephrine–adrenergic receptor signaling represents the most extensively characterized pathway (39, 61, 62). Cholinergic- and VIPergic-associated autonomic signals may also contribute, although the precise anatomical origin of these fibers remains incompletely resolved. By contrast, leptin, serotonin, endocannabinoids, dopamine, and the norepinephrine transporter should be regarded primarily as upstream, local, or receptor-dependent modulators rather than as equivalent transmitters released by bone-innervating sympathetic fibers. These pathways do not act as isolated signals; rather, they converge on bone-related cells and collectively shape the balance between osteogenesis, osteoclastogenesis, marrow adiposity, cartilage homeostasis, and repair responses (**Figure 3**).

### Norepinephrine–adrenergic receptor signaling in bone remodeling

Adrenergic signaling represents the classical route by which sympathetic nerves directly regulate skeletal remodeling (39, 62). Upon sympathetic activation, postganglionic fibers release norepinephrine (NE), the principal sympathetic neurotransmitter, into the local bone microenvironment. NE then acts on adrenergic receptors (ARs) expressed by bone-related cells, thereby transmitting sympathetic signals to the skeletal microenvironment (63, 64). ARs belong to the G protein-coupled receptor family and are generally classified into α- and β-adrenergic receptor subtypes. Although both receptor classes have been detected in bone-related cells, β-ARs predominate in skeletal tissue and are regarded as the major mediators of sympathetic regulation of bone metabolism (65).

The predominance of β-AR signaling is particularly evident in osteoblast-lineage cells. Osteoblasts express β1, β2, and β3-ARs, and β-AR expression changes dynamically during MSC-to-osteoblast differentiation (66). This dynamic receptor profile provides a cellular basis for stage-dependent responses of osteogenic lineage cells to sympathetic stimulation. Among these receptor subtypes, β2-AR has been most extensively implicated in the catabolic regulation of bone remodeling. In osteoblast-lineage cells and MSCs, β-AR activation suppresses osteogenic differentiation mainly through the cAMP/PKA/CREB pathway, thereby reducing osteoblast proliferation, differentiation, and mineralization (67–69). The inhibitory effect of β-AR signaling on bone formation is supported by both *in vivo* and *in vitro* evidence. *In vivo*, reduced catecholamine synthesis caused by dopamine β-hydroxylase deficiency results in a high-bone-mass phenotype, and pharmacological β-AR blockade with propranolol increases bone mass. Conversely, β-AR activation by isoproterenol or β2-selective agonists such as clenbuterol and salbutamol induces bone loss (70–72). These genetic and pharmacological findings indicate that enhanced catecholamine–β-AR signaling negatively regulates bone mass accrual. Consistently, *in vitro* studies show that high concentrations of NE suppress the proliferation and regenerative capacity of bone marrow mesenchymal stem cells through ERK1/2 and PKA phosphorylation (73). Together, these observations suggest that sustained NE–β-AR activation directly impairs osteoblast-lineage anabolic activity and shifts bone remodeling toward reduced bone formation. Recent cellular evidence further indicates that β2-adrenergic signaling alters osteoblast differentiation and migration through increased C-terminal phosphorylation of β-catenin at Ser552 and Ser675. This response was attenuated in β2-adrenergic receptor-deficient stromal cells, suggesting a receptor-dependent mechanism distinct from canonical Wnt-associated N-terminal β-catenin regulation (74).

In addition to suppressing bone formation, β2-AR signaling promotes bone resorption through bone cell-mediated regulation of osteoclastogenesis (75). In osteoblast-lineage cells, β2-AR activation promotes ATF4 phosphorylation and upregulates RANKL expression, thereby shifting the RANKL/OPG balance toward osteoclast differentiation and bone resorption (76, 77). Osteocyte-specific evidence further indicates that β2-AR signaling contributes to bone resorption by regulating osteoclastogenic mediators, including RANKL and OPG. Beyond the regulation of osteoclastogenic mediators, sympathetic signaling may directly promote osteocyte-mediated perilacunar remodeling. Guo et al. showed that norepinephrine activated β2-adrenergic receptors on osteocytes and promoted the release of extracellular vesicles containing matrix-degrading enzymes, whereas depletion of TH-positive sympathetic fibers, osteocyte-specific Adrb2 deletion, or β-adrenergic blockade attenuated cortical perilacunar resorption during lactation (78). Consistently, Adrb2-deficient mice exhibit a high-bone-mass phenotype, supporting the role of β2-AR signaling in restraining bone formation and promoting catabolic remodeling (77, 79, 80). Pharmacological studies also support this mechanism: isoproterenol increases osteopontin expression through β2-AR/cAMP signaling and contributes to bone loss, whereas selective β2-AR blockade with butoxamine ameliorates skeletal growth impairment and abnormal remodeling in a chronic intermittent hypoxia model, particularly in the mandibular condylar region (81, 82). Together, these findings indicate that the NE–β2-AR pathway exerts a dual catabolic effect on bone remodeling by suppressing osteoblast-lineage activity while enhancing RANKL-dependent osteoclastogenesis (76, 77, 83, 84).

Adrenergic regulation of skeletal remodeling is not restricted to β2-AR signaling. β1-AR and α-AR pathways may also participate in bone metabolism, although their effects appear to be more context-dependent. In normotensive and spontaneously hypertensive rat models, β1-AR rather than β2-AR has been implicated in the regulation of BMSC osteogenesis and mineralization, and clinical evidence also suggests a negative association between β1-AR activity and human bone metabolism (71, 85, 86). α-ARs may also contribute to skeletal regulation. α1-AR mRNA has been detected in osteoblast and osteoclast lineages (87, 88). Activation of α1-AR enhances MC3T3-E1 osteoblastic cell proliferation through Cebpd-related mechanisms, whereas systemic administration of the nonspecific α1-AR antagonist prazosin reduces bone formation. In addition, α1B-AR-deficient mice display reduced femoral cancellous bone mass without a marked bone-resorbing phenotype (87). α2C-AR signaling has also been linked to bone catabolism, as elevated thyroid hormone can interact with the sympathetic nervous system to induce bone loss through an α2C-AR-dependent pathway (89). These observations suggest that β2-AR is the dominant adrenergic mediator of sympathetic catabolic regulation in bone, whereas β1-AR and α-AR subtypes may modify skeletal outcomes under specific physiological or pathological conditions.

Overall, adrenergic signaling provides a direct molecular route by which sympathetic nerves regulate bone remodeling. Sustained NE–β2-AR activation generally favors bone loss by inhibiting osteoblast-lineage anabolic activity and promoting RANKL-dependent osteoclastogenesis (9, 79, 90). In contrast, β1-AR and α-AR pathways appear to exert more context-specific effects depending on receptor subtype, target cell population, endocrine status, mechanical environment, and disease stage.

### Neuropeptide Y signaling in osteogenesis, osteoclastogenesis, and bone–fat balance

Neuropeptide Y (NPY) is a highly conserved 36-amino-acid peptide and an important sympathetic co-transmitter involved in skeletal regulation. In peripheral sympathetic pathways, NPY is commonly co-localized and co-released with norepinephrine at sympathetic nerve terminals, and single-cell RNA sequencing of sympathetic ganglia has shown that a substantial proportion of sympathetic neuronal clusters express high levels of NPY (91–93). Through this close association with sympathetic innervation, NPY provides an additional neurochemical pathway by which neural stress responses and energy metabolism influence bone remodeling, marrow adiposity, and skeletal homeostasis (94, 95).

The biological effects of NPY are mediated by multiple G protein-coupled receptor subtypes, including Y1R, Y2R, Y4R, Y5R, and Y6R, among which Y1R and Y2R are most closely associated with skeletal metabolism (96). Y1R is generally regarded as the major peripheral receptor in bone and is expressed in osteoblast-lineage cells and osteocytes, whereas Y2R is more closely related to central hypothalamic regulation of bone formation (97, 98). Consistently, Npy1r mRNA has been detected in calvaria, long bone, and primary calvarial osteoblasts, whereas Npy2r mRNA is mainly restricted to the brain (99, 100). This receptor distribution indicates that NPY regulates bone through both peripheral and central pathways, with peripheral Y1R signaling providing a direct route for NPY to act on bone-forming cells.

In the peripheral skeletal microenvironment, NPY–Y1R signaling is closely involved in osteoblast activity and mesenchymal lineage allocation (101). Accumulating evidence indicates that excessive NPY signaling suppresses osteogenic differentiation and favors adipogenic commitment of mesenchymal stem/stromal cells. In calvarial osteoblasts, NPY treatment decreases late-stage osteogenic markers such as Bglap and markedly reduces mineral deposition (97). NPY also suppresses the osteogenic potential of BMSCs while supporting adipogenesis *in vivo*. Mechanistically, NPY–Y1R signaling downregulates osteogenic transcriptional regulators such as Tead1 and JunB through the cAMP/PKA/CREB pathway, thereby shifting mesenchymal lineage commitment from osteogenesis toward adipogenesis. In aging and osteoporosis, osteocytes tend to produce higher levels of NPY, further promoting the conversion of BMSCs from osteogenic to adipogenic differentiation and contributing to bone–fat imbalance (95).

Genetic and pharmacological evidence supports the negative regulatory role of peripheral NPY–Y1R signaling in bone mass accrual. Global NPY knockout increases bone mass throughout the skeleton and enhances osteoblast activity, whereas lineage-specific deletion of NPY1R in osteoblasts and osteocytes increases both cancellous and cortical bone mass (102). Similarly, NPY1R knockout mice show elevated bone mass, while conditional deletion of hypothalamic NPY1R does not markedly affect bone homeostasis, supporting a direct local role of peripheral NPY–Y1R signaling in bone-forming cells (103). Pharmacological evidence is consistent with this interpretation: local administration of an NPY1R antagonist increases tibial BMD in male Wistar rats, whereas NPY treatment reduces tibial BMD (104, 105). High NPY levels have also been linked to glucocorticoid-induced trabecular deterioration and marrow adiposity, while NPY deficiency preserves trabecular and cortical microarchitecture and protects against glucocorticoid-induced skeletal defects and marrow adiposity (106).

NPY signaling may also influence bone resorption, although its effects on osteoclast biology are more complex than its inhibitory effects on osteoblast-lineage cells. Loss of Y1R increases osteoblast-derived RANKL expression, thereby promoting osteoclast differentiation (107). In contrast, NPY treatment of BMSCs upregulates osteoprotegerin (OPG), which may inhibit RANKL-mediated osteoclastogenesis (108). In osteoclast-lineage cells, Npy1r expression increases during osteoclast differentiation. Germline deletion of NPY1R promotes the formation of large multinucleated osteoclasts through RANKL-induced MCP-1 overexpression, but these osteoclasts show impaired resorptive function, with reduced eroded area, pit formation, and expression of matrix degradation markers such as MMP9 and CTSK (107). These findings suggest that NPY–Y1R signaling can regulate both osteoclast formation and resorptive capacity, with the final effect depending on the responding cell type and local signaling context.

NPY should therefore not be interpreted as uniformly detrimental to bone metabolism. Exogenous NPY at low concentrations has been reported to increase the expression of osteogenic genes, including Alp, Runx2, and Col1a1, in BMSCs, and to enhance VEGF and BMP-2 expression through canonical Wnt signaling (109). NPY can also promote BMSC proliferation and protect against apoptosis through activation of the Wnt/β-catenin pathway (110). These findings suggest that NPY may support osteogenic and angiogenic responses under specific experimental or repair-related conditions (109, 111). Overall, NPY signaling exerts context-dependent effects on skeletal remodeling. Sustained or excessive peripheral NPY–Y1R activation generally suppresses osteoblast-lineage activity and promotes marrow adiposity, whereas low-dose or condition-specific NPY signaling may contribute to BMSC survival, osteogenic differentiation, and repair-associated bone formation.

### Sympathetic-associated cholinergic and VIPergic signaling in skeletal remodeling

In addition to classical noradrenergic signaling, bone-innervating autonomic fibers also include cholinergic and VIPergic components (50, 53, 112, 113). This component should be interpreted cautiously, because cholinergic regulation of bone has often been discussed in the context of parasympathetic signaling, and the precise origin of skeletal cholinergic fibers remains incompletely resolved (51, 53). Earlier tracing studies suggested a parasympathetic contribution, whereas more recent evidence indicates that at least a subset of skeletal cholinergic fibers may be sympathetic-associated (26, 45). Consistent with this possibility, neonatal ablation of sympathetic fibers reduces not only adrenergic fibers but also cholinergic fibers in bone, supporting the view that at least a subset of skeletal cholinergic innervation may be sympathetic-associated (53).

The functional significance of this cholinergic component has been increasingly recognized in direct skeletal regulation. Cholinergic innervation has been shown to support bone formation during postnatal growth and exercise, indicating that it may exert anabolic effects distinct from the catabolic actions commonly attributed to the NE–β2AR pathway (114). Mechanistically, cholinergic fibers interact with osteocytes through an NRTN–GFRα2-related neuro-osteocyte interface (114, 115). This interaction contributes to osteocyte survival, osteocyte network integrity, and bone formation, whereas loss of skeletal cholinergic fibers leads to osteocyte atrophy and reduced bone formation. These findings suggest that cholinergic signaling may participate directly in maintaining osteocyte function and skeletal anabolic activity.

VIPergic innervation represents another non-noradrenergic component of skeletal autonomic regulation (116). Although vasoactive intestinal peptide (VIP) is often discussed together with cholinergic or parasympathetic signaling, classic denervation studies demonstrated VIP-containing sympathetic nerve fibers in the periosteum and bone (50, 117). Functionally, VIP appears to support bone formation and repair. In sympathectomized mice, VIP administration improves fracture healing and partially counteracts the adverse skeletal effects of sympathetic denervation (118). In osteogenic cultures, VIP reduces the RANKL/OPG ratio, suggesting a direct regulatory effect on osteoblast–osteoclast coupling (119). VIP has also been reported to suppress osteoclast differentiation by attenuating NFATc1-related osteoclastogenic signaling (120).

Taken together, skeletal cholinergic and VIPergic signaling should be viewed as non-noradrenergic components associated with autonomic, and at least partly sympathetic, regulation of bone. Unlike the sustained NE–β2AR pathway, which generally favors bone loss by suppressing osteoblast-lineage activity and promoting osteoclastogenesis, cholinergic and VIPergic signaling may support osteocyte survival, bone formation, fracture repair, and restraint of osteoclast activation (121). Therefore, skeletal sympathetic regulation is functionally heterogeneous and includes both catabolic noradrenergic signaling and anabolic or repair-supportive non-noradrenergic components.

### Upstream and local modulators of sympathetic skeletal signaling

In addition to neurotransmitter- and receptor-defined pathways, sympathetic regulation of bone metabolism is shaped by upstream, local, and temporal modulatory mechanisms. These factors do not necessarily function as classical neurotransmitters released by bone-innervating sympathetic fibers. Instead, they regulate central sympathetic outflow, local neurotransmitter availability, cellular responsiveness to adrenergic signals, or the timing of sympathetic effects on skeletal tissues. In this context, circadian clocks, the norepinephrine transporter, leptin, serotonin-related pathways, the endocannabinoid system, and dopaminergic signaling influence the intensity, duration, timing, and skeletal consequences of sympathetic signaling.

### Circadian coupling of sympathetic signaling and bone remodeling

Circadian regulation represents a major temporal modulator of sympathetic control of skeletal homeostasis. The suprachiasmatic nucleus coordinates peripheral clocks through neural and endocrine outputs, while osteoblasts and osteoclasts contain intrinsic molecular clocks involving BMAL1/CLOCK, PER/CRY, and REV-ERB proteins. Early genetic studies linked these clock components to the leptin–sympathetic pathway: deletion of *Per* or *Cry* genes increased osteoblast proliferation and bone mass and disrupted leptin-dependent suppression of bone formation (122). These findings indicate that the molecular clock functions as an intracellular interface through which central sympathetic signals regulate osteoblast activity.

Adrenergic signaling can also entrain local clock-controlled programs in osteoblast-lineage cells. In human osteoblasts, adrenergic and glucocorticoid stimulation induces rhythmic expression of PER and BMAL1 genes (123). In murine osteoblasts, β-adrenergic stimulation regulated rhythmic Ptgs2 expression through NFIL3- and BMAL1/PER2-related mechanisms (124). α1-Adrenergic signaling similarly regulated the temporal expression of Bmp4 through NFIL3 and controlled circadian Tnfrsf11b expression through α1B-adrenergic receptor-dependent modulation of BMAL1, PER2, and REV-ERBα (125). Because Tnfrsf11b encodes osteoprotegerin, these pathways connect rhythmic adrenergic input not only to osteoblast function but also to the temporal regulation of RANKL–OPG-dependent osteoclastogenesis. Consistent with this concept, cell-specific Bmal1-deficient models demonstrate that disruption of peripheral skeletal clocks produces lineage- and compartment-dependent effects on trabecular bone formation, cortical bone maintenance, and osteoblast–osteoclast coupling (126–128).

Circadian regulation of skeletal remodeling is not mediated by sympathetic signaling alone. Osteoclastogenesis is also influenced by intrinsic clock components, glucocorticoid rhythms, feeding-related cues, and rhythmic signals derived from osteoblast-lineage cells. Notably, osteoclast-specific Bmal1 deletion has produced different skeletal phenotypes across experimental models, suggesting that the contribution of the autonomous osteoclast clock may depend on the targeting strategy, developmental stage, and experimental context. In humans, bone-turnover markers display marked daily variation, and recent constant-routine evidence indicates that circulating CTX retains an endogenous approximately 24-hour rhythm after major behavioral and environmental time cues are controlled (129). Experimental sleep restriction combined with circadian disruption also reduced P1NP and altered sclerostin or CTX concentrations in healthy men and women (130, 131), although these changes cannot be attributed exclusively to sympathetic activity. Beyond bone cells, rhythmic norepinephrine release regulates stromal CXCL12 expression and hematopoietic stem and progenitor cell trafficking through β3-adrenergic signaling (132). Together, these findings indicate that the skeletal consequences of sympathetic activity depend not only on signal intensity and receptor subtype, but also on circadian phase and the integrity of peripheral bone and marrow clocks.

### Norepinephrine transporter-mediated adrenergic signal termination

The norepinephrine transporter (NET) is a key regulator of adrenergic signal termination. By mediating the reuptake of synaptic NE, NET limits the duration of NE signaling and helps maintain local catecholamine homeostasis (133, 134). In bone, the biological effect of NE depends not only on its release from sympathetic fibers but also on its local clearance and availability around bone-related cells (60, 135). Therefore, NET provides an important mechanism by which the skeletal microenvironment may regulate the intensity and persistence of adrenergic signaling.

Evidence indicates that bone cells are involved in this local handling of NE. Osteoblasts and osteocytes can take up, but do not synthesize, NE through NET-dependent mechanisms, suggesting that these cells may participate in local NE clearance rather than serving as a source of catecholamine production (136). Consistently, differentiated osteoblasts show NET-mediated NE uptake *in vitro*, and NET-specific NE uptake has also been detected in cortical bone lacunae *in vivo* (133). These findings indicate that NET is not only a neuronal transporter but also a local regulator of NE availability within bone tissue.

The skeletal relevance of NET-mediated NE clearance is further supported by genetic evidence. Net-deficient mice exhibit impaired NE reuptake, reduced bone formation, increased bone resorption, lower peak bone mass, and reduced bone strength, despite low sympathetic outflow and elevated plasma NE (136). This phenotype suggests that defective NE clearance can prolong adrenergic exposure in the skeletal microenvironment and shift bone remodeling toward a catabolic state. Thus, NET functions as a local checkpoint that restrains excessive NE signaling and helps preserve the balance between bone formation and bone resorption.

However, the role of NET may differ between steady-state remodeling and bone repair. Systemic pharmacological NET inhibition does not appear to impair early or middle-stage femoral fracture healing and may even improve late-stage healing outcomes (137). These findings suggest that NET-dependent regulation of NE availability is context-dependent. During steady-state remodeling, impaired NET activity may enhance adrenergic catabolic signaling and reduce bone mass, whereas during regenerative repair, transient changes in NE handling may have stage-specific effects. Therefore, NET should be viewed as an important modulator of adrenergic signal duration whose skeletal consequences depend on the physiological or reparative context.

### Leptin–SNS axis in neuroendocrine skeletal regulation

Leptin is an adipocyte-derived hormone that links energy metabolism with skeletal remodeling through both central and peripheral mechanisms (138). In the context of sympathetic skeletal regulation, leptin should not be regarded as a classical neurotransmitter released by sympathetic nerve fibers. Rather, it functions as an upstream neuroendocrine regulator that modulates sympathetic output to bone. Centrally, leptin acts on hypothalamic neural circuits to increase sympathetic outflow to bone. Norepinephrine released from sympathetic nerve terminals subsequently activates β2-adrenergic receptors on osteoblast-lineage cells, thereby suppressing bone formation (49).

This leptin–SNS pathway provides an important mechanism by which systemic energy status is coupled to bone formation. The central leptin–SNS pathway represents one of the foundational demonstrations of neural control over bone mass. Following the identification of a hypothalamic relay through which leptin suppresses bone formation, Takeda et al. established the sympathetic nervous system as the principal peripheral effector of this pathway. They showed that reduced sympathetic tone contributed to the high-bone-mass phenotype of leptin-deficient mice, whereas interruption of β-adrenergic signaling prevented central leptin from suppressing bone formation. Conversely, β-adrenergic stimulation reduced bone mass, while β-blockade increased bone mass independently of major changes in body weight (49). These findings support the concept that leptin can regulate bone remodeling indirectly through hypothalamic activation of sympathetic adrenergic signaling.

In parallel with its central SNS-dependent pathway, leptin can also act directly on peripheral skeletal cells through leptin receptor (LepR) expressed by mesenchymal stem/stromal cells, osteoblasts, osteoclasts, and osteocytes (139, 140). Peripheral leptin/LepR signaling activates downstream pathways such as JAK/STAT, PI3K/Akt, and MAPK, thereby influencing osteoblast proliferation and differentiation, osteoclast activity, and bone remodeling (141). In several experimental settings, leptin promotes osteoblast proliferation and osteogenic differentiation, regulates the OPG/RANKL balance, and suppresses osteoclastogenesis (142, 143). LepR-positive mesenchymal stem/stromal cells also represent an important cellular source for bone formation and skeletal repair, further supporting the direct role of peripheral leptin signaling in the skeletal microenvironment (139).

Therefore, leptin regulates bone metabolism through two partially opposing modes of action. The central leptin–SNS pathway tends to suppress bone formation by enhancing sympathetic adrenergic signaling, whereas peripheral leptin signaling may support osteoblast activity and restrain bone resorption under specific conditions. This dual pattern explains why leptin should be viewed as a neuroendocrine modulator of sympathetic skeletal signaling rather than as a uniformly anabolic or catabolic factor. Its skeletal effects depend on the balance between central sympathetic activation and peripheral LepR-mediated actions, as well as leptin concentration, exposure duration, target cell type, and physiological or pathological context.

### Serotonin-related regulation of sympathetic tone and bone metabolism

Serotonin, or 5-hydroxytryptamine (5-HT), is involved in skeletal regulation in a source-dependent manner. In the context of sympathetic skeletal signaling, brain-derived serotonin is particularly relevant because it can regulate bone metabolism through central neural circuits that control sympathetic outflow. Within the central nervous system, serotonin provides excitatory input to hypothalamic neurons, which subsequently inhibit sympathetic preganglionic neurons. This reduction in sympathetic flow decreases sympathetic restraint on bone formation and thereby favors bone mass accrual. Consistently, mice lacking tryptophan hydroxylase 2, the rate-limiting enzyme for brain serotonin synthesis, display low bone mass accompanied by reduced bone formation (144). These findings indicate that brain-derived serotonin can support bone formation indirectly by suppressing sympathetic tone.

The skeletal effects of serotonin differ when it acts outside the central sympathetic pathway. In contrast to brain-derived serotonin, peripheral serotonin can exert negative local effects on bone by disrupting matrix assembly and mineralization (145). Peripheral serotonin may also enhance bone resorption, as serotonin has been reported to amplify RANKL-induced osteoclastogenesis *in vitro* (146–148). Therefore, serotonin should not be interpreted as a uniform regulator of bone metabolism. Its skeletal effects depend on its source and site of action: brain-derived serotonin may favor bone formation by reducing sympathetic outflow, whereas peripheral serotonin may impair bone formation and promote osteoclastogenesis through local bone-cell-related mechanisms.

### Endocannabinoid modulation of sympathetic neurotransmission

The endocannabinoid system provides another modulatory mechanism in sympathetic skeletal regulation. This system consists of endogenous ligands, such as 2-arachidonoylglycerol (2-AG), cannabinoid receptors, and enzymes involved in endocannabinoid synthesis and degradation. Cannabinoid CB1 receptors are distributed mainly in the central and peripheral nervous systems, whereas CB2 receptors are predominantly expressed in peripheral tissues, including the skeletal system (149, 150). This receptor distribution indicates that endocannabinoid signaling may influence bone metabolism through both neural and local skeletal mechanisms.

In the context of sympathetic regulation, CB1 is particularly relevant because it can modulate norepinephrine (NE) release from sympathetic nerve endings. Bone-cell-derived 2-AG can activate CB1 receptors on sympathetic terminals and restrain NE release through retrograde signaling (150, 151). Through this mechanism, CB1 signaling may regulate the strength of sympathetic adrenergic input to bone. Consistently, sympathetic CB1 receptor deficiency in aged mice increases bone formation and reduces osteoclastogenesis, suggesting that CB1-dependent regulation of sympathetic neurotransmission contributes to age-related skeletal remodeling (152). However, CB1-related skeletal phenotypes may vary according to mouse strain, genotype, sex, and age, indicating that its effect on bone mass is context-dependent.

In contrast to CB1, CB2 signaling is more directly associated with local skeletal remodeling. Activation of CB2 receptors promotes bone formation and attenuates bone resorption, whereas CB2-deficient mice initially acquire normal peak trabecular bone mass but later develop low bone mass due to accelerated bone turnover (153, 154). These findings indicate that CB2 participates in the maintenance of skeletal homeostasis by regulating the balance between bone formation and bone resorption.

Taken together, the endocannabinoid system influences bone metabolism through two related but distinct mechanisms. CB1 signaling modulates sympathetic neurotransmission by regulating NE release from sympathetic nerve endings, whereas CB2 signaling acts more directly within the skeletal microenvironment to promote bone formation and restrain bone resorption. Therefore, endocannabinoid signaling should be viewed as a context-dependent modulator of sympathetic skeletal regulation rather than as a classical sympathetic neurotransmitter pathway.

### Dopaminergic modulation of osteogenesis and osteoclastogenesis

Dopaminergic signaling may also participate in sympathetic-related skeletal regulation, although it should be distinguished from the classical norepinephrine (NE)–adrenergic receptor axis (155). Dopamine is a catecholamine neurotransmitter and neuromodulator closely associated with sympathetic neurobiology (156). However, current evidence in bone metabolism mainly supports receptor-mediated actions of dopamine on bone-related cells, rather than a well-established mechanism in which bone-innervating sympathetic fibers release dopamine as a principal skeletal effector. Therefore, dopamine is better considered a complementary catecholaminergic pathway that may modulate bone remodeling in parallel with, rather than as a substitute for, NE-mediated sympathetic signaling.

Dopamine exerts its biological effects through two major G protein-coupled receptor subfamilies: D1-like receptors, including D1R and D5R, and D2-like receptors, including D2R, D3R, and D4R (157). These receptors have been detected in osteoblasts and osteoclasts, providing a cellular basis for dopamine-mediated regulation of both bone formation and bone resorption (157, 158). In osteoblast-lineage cells, D1R activation promotes osteogenic differentiation and mineralization through the cAMP/PKA signaling pathway, enhances ERK1/2 phosphorylation, and upregulates osteogenic markers such as RUNX2, alkaline phosphatase (ALP), and osteopontin (OPN) (157). These findings indicate that D1R-mediated dopaminergic signaling may support osteoblast activity and bone formation.

In contrast, D2R signaling is more closely related to the regulation of osteoclast differentiation. Activation of D2R suppresses osteoclastogenesis through the cAMP/PKA/CREB pathway and RANK-related signaling (158). Thus, dopamine may contribute to skeletal homeostasis through receptor-specific actions: D1R signaling promotes osteoblast-associated bone formation, whereas D2R signaling restrains osteoclast differentiation. Taken together, dopaminergic signaling should be viewed as a modulatory catecholaminergic pathway that directly affects osteogenesis and osteoclastogenesis, while remaining distinct from the classical NE–adrenergic pathway of sympathetic skeletal regulation.

## Osteoimmune mechanisms of sympathetic regulation in bone remodeling

**Figure 4:**
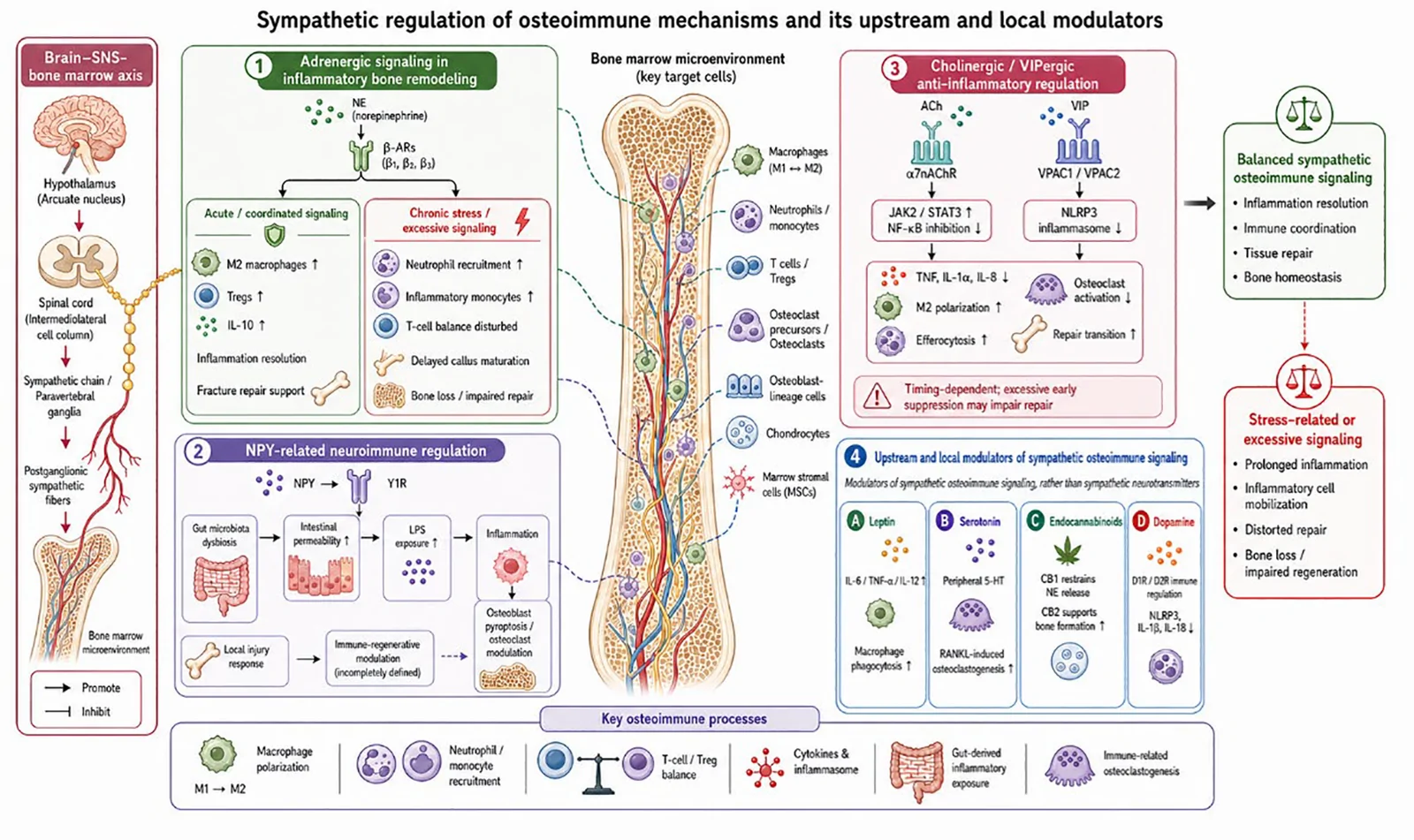
Sympathetic regulation of osteoimmune mechanisms and its upstream and local modulators. Sympathetic osteoimmune regulation links central sympathetic output with immune and skeletal remodeling within the bone marrow microenvironment, while additional upstream and local pathways modulate the intensity, duration, and context of these responses. Signals from the brain–SNS–bone marrow axis modulate macrophages, neutrophils/monocytes, T cells/Tregs, osteoblast-lineage cells, osteoclasts, chondrocytes, and marrow stromal cells. (1) Adrenergic signaling exerts context-dependent effects on inflammatory bone remodeling, supporting inflammation resolution and repair under acute or coordinated activation, but promoting chronic inflammation, bone loss, and impaired repair during excessive or prolonged activation. (2) NPY-related neuroimmune signaling connects sympathetic activity with gut-derived inflammatory exposure, macrophage/osteoclast activation, and local immune-regenerative responses. (3) Cholinergic and VIPergic sympathetic-associated pathways act as anti-inflammatory regulators by limiting cytokine production, inflammasome activation, immune-driven osteoclastogenesis, and excessive early inflammation during repair. (4) Leptin, serotonin, endocannabinoid signaling, and dopaminergic signaling act as upstream or local modulators of sympathetic osteoimmune signaling rather than as equivalent neurotransmitters released from bone-innervating sympathetic fibers. These pathways further influence macrophage polarization and phagocytosis, osteoclastogenesis, cytokine signaling, inflammasome activity, and inflammatory bone remodeling.

The osteoimmune function of sympathetic signaling differs from its direct skeletal function (26, 60). In direct skeletal regulation, sympathetic signals alter osteogenic differentiation, osteoclastogenesis, marrow adiposity, cartilage metabolism, or vascular coupling by acting on bone-related cells. In osteoimmune regulation, sympathetic signals influence bone remodeling indirectly or jointly by changing the immune context in which bone cells operate. This includes regulation of neutrophil recruitment, macrophage phenotype switching, T-cell populations, anti-inflammatory cytokine production, inflammasome activation, gut-derived inflammatory exposure, and osteoclast precursor behavior (**Figure 4**) (159). Recent evidence further indicates that central neuronal circuits can regulate the bone marrow immune niche through sympathetic noradrenergic output. In experimental autoimmune encephalomyelitis, hypothalamic AgRP neurons modulated norepinephrine and β3-adrenergic signaling in the bone marrow and thymus, thereby influencing myeloid hematopoiesis and Treg-related immune homeostasis, although direct skeletal consequences were not assessed (160).

This distinction is critical because the same sympathetic-related pathway may have different skeletal consequences depending on inflammatory stage and disease context. In physiological bone repair, transient inflammation is necessary, and sympathetic activity may contribute to an anti-inflammatory, pro-reparative environment (161, 162). For example, traumatic brain injury combined with fracture has been reported to enhance hypothalamic regulation of sympathetic activity, increase M2 macrophage infiltration in callus tissue, stimulate anti-inflammatory myeloid cell expansion in bone marrow, and elevate serum IL-10, thereby supporting fracture healing (163). In contrast, chronic psychological stress or post-traumatic stress disorder can activate sympathetic catecholamine signaling, promote neutrophil and inflammatory monocyte mobilization, increase neutrophil infiltration into fracture hematoma, and impair callus formation; these effects can be ameliorated by β-adrenergic blockade (164–166). Thus, sympathetic osteoimmune regulation is highly stage-dependent: properly coordinated sympathetic signaling may support inflammation resolution, whereas sustained stress-related sympathetic activation may prolong or distort inflammation and impair bone repair.

Therefore, the following sections discuss sympathetic osteoimmune mechanisms from the perspective of inflammatory bone remodeling rather than repeating direct effects on osteoblasts and osteoclasts. The emphasis is placed on immune-cell recruitment, macrophage polarization, T-cell regulation, cytokine production, inflammasome signaling, and immune-derived osteoclastogenesis.

### Adrenergic regulation of inflammatory bone remodeling

Adrenergic signaling is the classical effector pathway of sympathetic activity. In the osteoimmune context, its relevance lies not in the direct regulation of osteoblasts or osteoclasts alone, but in the ability of catecholamines to modulate immune-cell dynamics during bone remodeling and repair. Norepinephrine (NE) released from sympathetic fibers can act on adrenergic receptors expressed by immune cells and stromal components within the bone marrow and fracture microenvironment (159, 167). Through this pathway, sympathetic activity may influence inflammatory cell mobilization, macrophage phenotype, T-cell composition, cytokine production, and the osteogenic immune environment. The functional interaction between sympathetic nerves and bone marrow stromal cells was demonstrated by Katayama et al., who showed that intact adrenergic neurotransmission was required for granulocyte colony-stimulating factor-induced hematopoietic stem and progenitor cell mobilization. Disruption of neural or adrenergic signaling prevented the normal suppression of osteoblast activity and downregulation of bone marrow CXCL12, thereby impairing progenitor-cell egress, whereas β2-adrenergic stimulation enhanced mobilization (168). Under physiological conditions, Méndez-Ferrer et al. further demonstrated that rhythmic norepinephrine release from sympathetic nerves induced circadian changes in stromal CXCL12 expression and hematopoietic stem and progenitor cell release through β3-adrenergic signaling (132).

Under certain regenerative conditions, coordinated sympathetic activation appears to support an anti-inflammatory microenvironment favorable for bone repair. Sympathetic nervous system activation has been associated with increased CD4^+^CD25^+^FoxP3^+^ regulatory T cells in bone marrow (169). In traumatic brain injury combined with fracture, increased hypothalamic regulation of sympathetic activity promotes M2 macrophage infiltration in the callus and stimulates anti-inflammatory myeloid populations in bone marrow, including Ly6_C_ monocytes and CD206^+^ macrophages. This response is accompanied by increased serum IL-10, suggesting that sympathetic output can contribute to inflammation resolution and fracture healing (163). β2-adrenergic receptors have also been implicated in M2 macrophage polarization, whereas β3-adrenergic receptors influence hematopoietic progenitor cell proliferation (165). The long-term integrity of this neural regulation is also relevant to aging, as Maryanovich et al. showed that degeneration of bone marrow adrenergic nerves and reduced β3-adrenergic signaling accelerated hematopoietic stem-cell aging, whereas selective β3-adrenergic stimulation improved the functional capacity of aged hematopoietic stem cells (170). These findings indicate that adrenergic signaling may promote bone repair by shaping immune-cell behavior within the osteogenic microenvironment. Recent single-cell evidence further showed that sympathetic inhibition enhanced calvarial defect repair by altering local immune cells, vascular structures, and mesenchymal populations, including increased type H vessel formation and the emergence of a macrophage population with senescence-associated features. Pharmacological clearance of senescent cells attenuated this regenerative effect, suggesting that senescent-like macrophages may transiently support osteogenesis and angiogenesis in a site- and injury-specific context (171).

The effect of adrenergic signaling, however, depends strongly on the duration and context of sympathetic activation. In post-traumatic stress disorder models, psychological stress increases neutrophil recruitment in bone marrow during the early phase of fracture healing and enhances neutrophil infiltration into fracture hematomas. These changes are accompanied by reduced CD4+ and CD8+ T-cell populations and impaired late-stage callus formation; propranolol treatment partly reverses these abnormalities, supporting the involvement of β-adrenergic signaling (164). Chronic stress can also activate sympathetic nerves to release NE, which acts on β3-adrenergic receptors in bone marrow cells and suppresses CXCL12 secretion, thereby promoting the egress of neutrophils and inflammatory monocytes into the circulation (165). In fracture hematomas, neutrophil-derived catecholamines may further activate α- and β-adrenergic receptors on chondrocytes and promote additional neutrophil recruitment through paracrine mechanisms, potentially involving CXCL1 released by mast cells and macrophages (166). This catecholamine-driven feed-forward inflammatory response may disturb endochondral ossification and impair bone regeneration. However, neutrophil adrenergic signaling is not uniformly detrimental during bone repair. Neutrophil-specific deletion of Adrb2 reduced early neutrophil recruitment and impaired callus bone formation in both non-osteoporotic and osteoporotic mice, indicating that temporally coordinated β2-adrenergic signaling is required for an effective early inflammatory response (172).

Adrenergic signaling also affects adaptive immune components during fracture repair. In ovariectomized fracture models, sympathectomy reduces CD8^+^ cytotoxic T cells and CD4^+^ helper T cells in the fracture hematoma, while macrophage numbers remain relatively unchanged (173). Because CD4^+^ T cells can contribute to bone remodeling by producing osteoprotegerin, the reduction in CD4^+^ T cells during early fracture healing may partly explain delayed callus maturation after sympathectomy. This observation suggests that sympathetic innervation does not simply suppress inflammation; rather, it helps maintain an appropriate immune-cell composition during the transition from inflammation to repair.

Taken together, adrenergic osteoimmune regulation is context-dependent. Acute and coordinated sympathetic activation may support fracture repair by promoting M2 macrophage infiltration, anti-inflammatory myeloid expansion, IL-10 production, and Treg-related immune regulation. In contrast, chronic stress-related adrenergic activation may mobilize neutrophils and inflammatory monocytes, enhance catecholamine-driven immune-cell recruitment, disturb T-cell balance, and delay callus maturation. This duality explains why β-adrenergic blockade can be protective in stress-impaired fracture healing, whereas complete loss of sympathetic input may also disrupt immune coordination during bone repair.

### NPY-related neuroimmune regulation in bone remodeling

Neuropeptide Y (NPY) is a sympathetic co-transmitter and stress-related neuropeptide involved in skeletal regulation (92, 93, 96). Its direct effects on osteoblast activity, mesenchymal stem/stromal cell fate, marrow adiposity, and bone–fat balance have been discussed in Section 3. In the osteoimmune context, NPY is more appropriately considered as a mediator linking sympathetic stress-related signaling with inflammatory exposure, immune-cell-related bone resorption, and osteoporotic bone loss.

One representative mechanism involves NPY-mediated alterations in the gut microbiota and their effects on bone metabolism. In postmenopausal osteoporosis, elevated NPY signaling aggravates bone loss by reshaping gut microbiota, increasing intestinal permeability, and enhancing systemic lipopolysaccharide (LPS) exposure (174). LPS-driven inflammatory stimulation can induce osteoblast pyroptosis, impair osteogenic differentiation, and further accelerate bone deterioration. Blockade of Y1R signaling attenuates these effects, suggesting that NPY–Y1R signaling may connect sympathetic/neuropeptide activity with gut-derived inflammatory skeletal damage. This mechanism indicates that NPY contributes to bone loss not only through direct inhibition of bone-forming cells, but also through an inflammation-mediated pathway.

As discussed in the section on direct skeletal regulation, NPY–Y1R signaling exerts complex and cell-dependent effects on osteoclast differentiation and resorptive function. Because osteoclasts arise from monocyte/macrophage-lineage precursors, these findings may also have osteoimmune relevance. However, the available evidence mainly concerns the regulation of osteoclastogenesis and mature osteoclast function, rather than direct effects of NPY on broader immune-cell populations. Therefore, the contribution of NPY signaling to osteoimmune remodeling should be interpreted cautiously and distinguished from its better-established direct effects on osteoblast-lineage cells and osteoclasts.

Evidence from bone injury models further suggests that NPY signaling may participate in the local inflammatory response during bone regeneration. In a femoral defect model, NPY and Y1R expression increases during the inflammatory phase, whereas this response is not observed in sham-operated animals (175). Immunohistochemical analysis indicates that polymorphonuclear cells recruited to the defect site are the major cells expressing both NPY and Y1R during this phase, suggesting a potential autocrine or local inflammatory role of the NPY system. Notably, NPY-positive nerve fibers are not clearly observed within the defect area, but are distributed along blood vessels in bone tissue and bone marrow, and increased NPY expression has also been detected in dorsal root ganglia. These observations indicate that NPY signaling is activated during the inflammatory phase of bone repair, but its cellular source and functional contribution to the osteoimmune microenvironment require further clarification.

Overall, NPY should be described cautiously as a sympathetic stress-related neuropeptide with potential osteoimmune functions. Current evidence suggests that NPY may influence inflammatory bone loss through gut-derived LPS exposure, osteoclast-lineage functional maturation, and local injury-associated inflammatory responses. However, compared with neuroimmune mediators such as CGRP, substance P, VIP, acetylcholine, and NGF, the immune mechanisms of NPY during bone remodeling and regeneration remain less well defined. Further studies are needed to clarify how NPY/Y1R signaling affects macrophages, neutrophils, T cells, and other immune populations during inflammatory bone loss and bone repair.

### Cholinergic and VIPergic regulation of inflammatory responses and osteoclast activation

In addition to the classical noradrenergic pathway, cholinergic and VIPergic signaling may also participate in osteoimmune regulation. As discussed in Section 3.3, skeletal autonomic innervation includes cholinergic and VIPergic components, although their anatomical origin should be interpreted cautiously because cholinergic regulation of bone has often been discussed in the context of parasympathetic signaling. Nevertheless, evidence supporting sympathetic-associated cholinergic fibers and VIP-containing sympathetic fibers in the periosteum and bone suggests that these non-noradrenergic pathways may contribute to autonomic regulation of the skeletal microenvironment (50, 53, 112, 113). In the osteoimmune context, their relevance lies mainly in the regulation of inflammatory cytokine production, macrophage phenotype, immune-cell infiltration, and immune-related osteoclast activation.

Acetylcholine (ACh) is a key mediator of cholinergic anti-inflammatory signaling. In systemic inflammatory models, efferent vagus nerve stimulation activates catecholaminergic splenic nerves and increases norepinephrine release. Norepinephrine then acts on β2-adrenergic receptors expressed by choline acetyltransferase-positive T cells, enhancing ACh secretion. ACh subsequently binds to α7 nicotinic acetylcholine receptors (α7nAChR) on macrophages and suppresses the production of pro-inflammatory cytokines, including TNF, IL-1α, and IL-8 (176–178). Although direct evidence for this complete pathway in bone repair remains limited, it provides an important autonomic immune framework through which cholinergic signaling may restrain excessive inflammation during skeletal repair.

The immunomodulatory effect of ACh is further supported by studies in tissue repair models. ACh can inhibit TNF production in lipopolysaccharide-stimulated macrophages, suppress NF-κB nuclear translocation, activate α7nAChR-mediated JAK2/STAT3 signaling, and inhibit mitochondrial DNA release and inflammasome activation (179–184). These mechanisms indicate that cholinergic signaling can limit macrophage-derived inflammatory responses. However, in bone regeneration, cholinergic enhancement may be context- and stage-dependent. In a rat tibial defect model, administration of the acetylcholinesterase inhibitor donepezil impaired bone regeneration and was accompanied by reduced macrophage and T-cell infiltration two weeks after injury (185). This finding suggests that appropriate immune-cell recruitment remains necessary for bone repair, and that excessive or premature suppression of inflammation may disturb the transition from injury response to regeneration.

VIPergic signaling represents another non-noradrenergic pathway involved in osteoimmune regulation. Vasoactive intestinal peptide (VIP) can be released from autonomic nerves and can also be produced by immune cells. It mainly acts through VPAC1 and VPAC2 receptors and has potent immunomodulatory properties (186, 187). In tendon repair, VIP suppresses macrophage-derived inflammatory mediators, promotes M2 macrophage polarization, and enhances macrophage efferocytosis by downregulating NF-κB signaling (188). In a mouse tooth extraction model, exogenous VIP promotes M2 macrophage polarization, decreases pro-inflammatory cytokines such as IL-1β, IL-6, and TNF, and increases anti-inflammatory or regulatory mediators such as IL-10 and TGF-β1 (189). These findings suggest that VIP can regulate the balance between inflammatory and reparative macrophage phenotypes.

The effect of VIP in bone repair also appears to depend on timing and baseline physiological conditions. During alveolar bone repair, VIP is naturally expressed and is associated with osteogenic factor expression, but additional exogenous VIP has only limited effects on bone healing under homeostatic conditions (189). This suggests that endogenous VIP signaling may already contribute to the physiological regulation of early inflammation and repair, whereas further activation does not necessarily enhance regeneration. Therefore, VIP should be viewed as an immunomodulatory neuropeptide that fine-tunes macrophage polarization, cytokine balance, and the resolution of local inflammatory responses rather than as a uniformly pro-regenerative factor.

Taken together, cholinergic and VIPergic pathways contribute to osteoimmune regulation mainly by shaping the inflammatory microenvironment during bone remodeling and repair. Their effects involve α7nAChR-mediated cytokine suppression, NF-κB/JAK2/STAT3-related inflammatory regulation, macrophage polarization, immune-cell infiltration, and the balance between inflammatory and reparative immune states. These mechanisms suggest that cholinergic and VIPergic signaling may serve as counter-regulatory components of autonomic control in inflammatory bone remodeling, but their effects depend strongly on timing, tissue context, and the level of endogenous signaling.

### Upstream and local immunomodulators of sympathetic osteoimmune regulation

Beyond classical sympathetic neurotransmitters and co-transmitters, several upstream and local mediators can modulate sympathetic osteoimmune regulation. Leptin, serotonin, endocannabinoids, and dopamine are not equivalent to NE as direct sympathetic neurotransmitters released from bone-innervating sympathetic fibers (26, 60). Instead, they influence sympathetic skeletal regulation by shaping immune-cell activation, inflammatory cytokine production, inflammasome signaling, gut-derived inflammatory exposure, and immune-related osteoclastogenesis.

#### Leptin as an immunometabolic amplifier of sympathetic osteoimmune regulation

Leptin is an adipocyte-derived hormone that links energy metabolism, hypothalamic sympathetic output, immune activation, and skeletal remodeling (138, 190, 191). Its central leptin–SNS pathway and peripheral skeletal actions have been discussed in Section 3. In the osteoimmune context, leptin is particularly relevant as a pleiotropic cytokine-like adipokine that connects metabolic status with inflammatory bone and joint remodeling.

Leptin participates in both innate and adaptive immune responses. It can stimulate the production of pro-inflammatory cytokines, including IL-6, TNF-α, and IL-12, enhance macrophage phagocytic activity, and promote natural killer cell proliferation (192–194). These immune effects may amplify inflammatory signaling within the skeletal microenvironment and create conditions that favor osteoclast activation and inflammatory bone loss. In this way, leptin provides a mechanistic link among adiposity, metabolic stress, sympathetic tone, and inflammation-associated skeletal remodeling.

Leptin is also involved in inflammatory cartilage and joint remodeling. In chondrocytes, leptin induces the production of IL-6, IL-8, nitric oxide, and prostaglandins, while upregulating matrix metalloproteinases and promoting extracellular matrix degradation (195, 196). These effects connect adipokine-driven immune-metabolic signaling with osteoarthritis-related cartilage catabolism, synovial inflammation, and subchondral bone remodeling. Therefore, leptin can be viewed as an immunometabolic amplifier within sympathetic skeletal regulation, integrating adiposity, hypothalamic–SNS activity, cytokine production, macrophage and natural killer cell responses, and inflammatory joint degeneration.

#### Serotonin-related inflammatory modulation in bone remodeling

Serotonin-related skeletal regulation is strongly source-dependent. Brain-derived serotonin can reduce sympathetic outflow and favor bone accrual, whereas peripheral serotonin may exert local negative effects on bone matrix formation and mineralization (144, 148). Because the central regulation of sympathetic outflow by serotonin has been discussed in Section 3, the osteoimmune relevance of serotonin mainly concerns peripheral serotonergic signaling and its potential contribution to inflammatory bone resorption.

Peripheral serotonin can impair osteoblast matrix assembly and mineralization (145). In the context of osteoimmune remodeling, serotonin has also been reported to enhance RANKL-induced osteoclastogenesis *in vitro* (146, 147). Since osteoclasts are derived from monocyte/macrophage-lineage precursors and RANKL is a central mediator of inflammatory bone resorption, this finding suggests that peripheral serotonergic signaling may amplify osteoclast differentiation under pro-resorptive inflammatory conditions. Therefore, serotonin may contribute to inflammatory bone remodeling not only by affecting bone-forming cells, but also by strengthening RANKL-dependent osteoclastogenesis.

The skeletal effects of serotonin-reuptake inhibition further illustrate the complexity of this pathway, because central and peripheral serotonergic actions may produce divergent outcomes (197). Thus, serotonin should not be described as a uniformly anabolic or catabolic factor. Rather, it is better viewed as a source-dependent regulator whose osteoimmune significance is mainly related to peripheral serotonin-mediated matrix impairment and RANKL-dependent osteoclastogenesis.

#### Endocannabinoid regulation of osteoimmune remodeling

The endocannabinoid system links neural regulation with skeletal and immune-related remodeling. This system consists of endogenous ligands, cannabinoid receptors, and enzymes involved in endocannabinoid synthesis and degradation. CB1 receptors are distributed mainly in the central and peripheral nervous systems, whereas CB2 receptors are predominantly expressed in peripheral tissues, including the skeletal and immune systems (149, 151, 198). This receptor distribution suggests that endocannabinoid signaling may influence osteoimmune remodeling through both neural and local peripheral mechanisms.

From the perspective of sympathetic regulation, CB1 is mainly relevant to norepinephrine (NE) release. Bone-cell-derived 2-arachidonoylglycerol can activate CB1 receptors on sympathetic nerve endings and restrain NE release through retrograde signaling (150, 151). By modulating local sympathetic adrenergic tone, CB1 signaling may indirectly affect inflammation-associated bone remodeling. However, the osteoimmune relevance of the endocannabinoid system is more closely related to CB2. CB2 activation promotes bone formation and attenuates bone resorption, whereas CB2 deficiency leads to accelerated bone turnover and age-related bone loss (150, 199). Because CB2 is expressed in peripheral skeletal and immune compartments, this pathway may participate in inflammatory bone remodeling by regulating osteoclast activity and the local immune-associated environment.

Therefore, endocannabinoid signaling should not be viewed as a direct equivalent of the NE–adrenergic receptor pathway. Rather, it functions as a modulatory system that fine-tunes sympathetic neurotransmission through CB1 and contributes to peripheral osteoimmune remodeling mainly through CB2. Its skeletal effects are context-dependent and may vary according to receptor subtype, cellular target, age, and local inflammatory status.

#### Dopaminergic control of inflammatory signaling in bone-related disorders

Dopamine is closely related to sympathetic neurobiology, but it should be distinguished from the classical norepinephrine (NE)–adrenergic receptor pathway in bone (156). Its receptor-mediated effects on osteoblasts and osteoclasts have been discussed in Section 3 (155, 200). In the osteoimmune context, dopamine is more appropriately considered as a complementary catecholaminergic pathway that modulates inflammatory signaling associated with bone-related disorders.

Dopamine regulates immune-cell activation, suppression, and proliferation mainly through D1R- and D2R-related pathways (201, 202). These immunoregulatory effects may influence the inflammatory microenvironment that contributes to bone and joint damage. For example, dopamine can inhibit NLRP3 inflammasome activation and reduce the production of pro-inflammatory cytokines, including IL-1β and IL-18 (203). Dopamine can also suppress systemic inflammatory mediators such as TNF-α and IL-6 through central or peripheral pathways. Through these mechanisms, dopaminergic signaling may attenuate inflammatory responses that otherwise promote osteoclast activation, joint inflammation, and tissue damage.

Therefore, dopamine should not be presented as a direct equivalent of NE-mediated sympathetic signaling in bone. Rather, it may function as a catecholaminergic osteoimmune modulator that links neural regulation with inflammatory signaling. Its relevance to bone-related disorders lies mainly in the regulation of immune-cell activity, inflammasome activation, and pro-inflammatory cytokine production, which may indirectly affect inflammatory bone remodeling and joint degeneration.

## Pathophysiological mechanisms of sympathetic dysregulation in bone-related disorders

**Figure 5:**
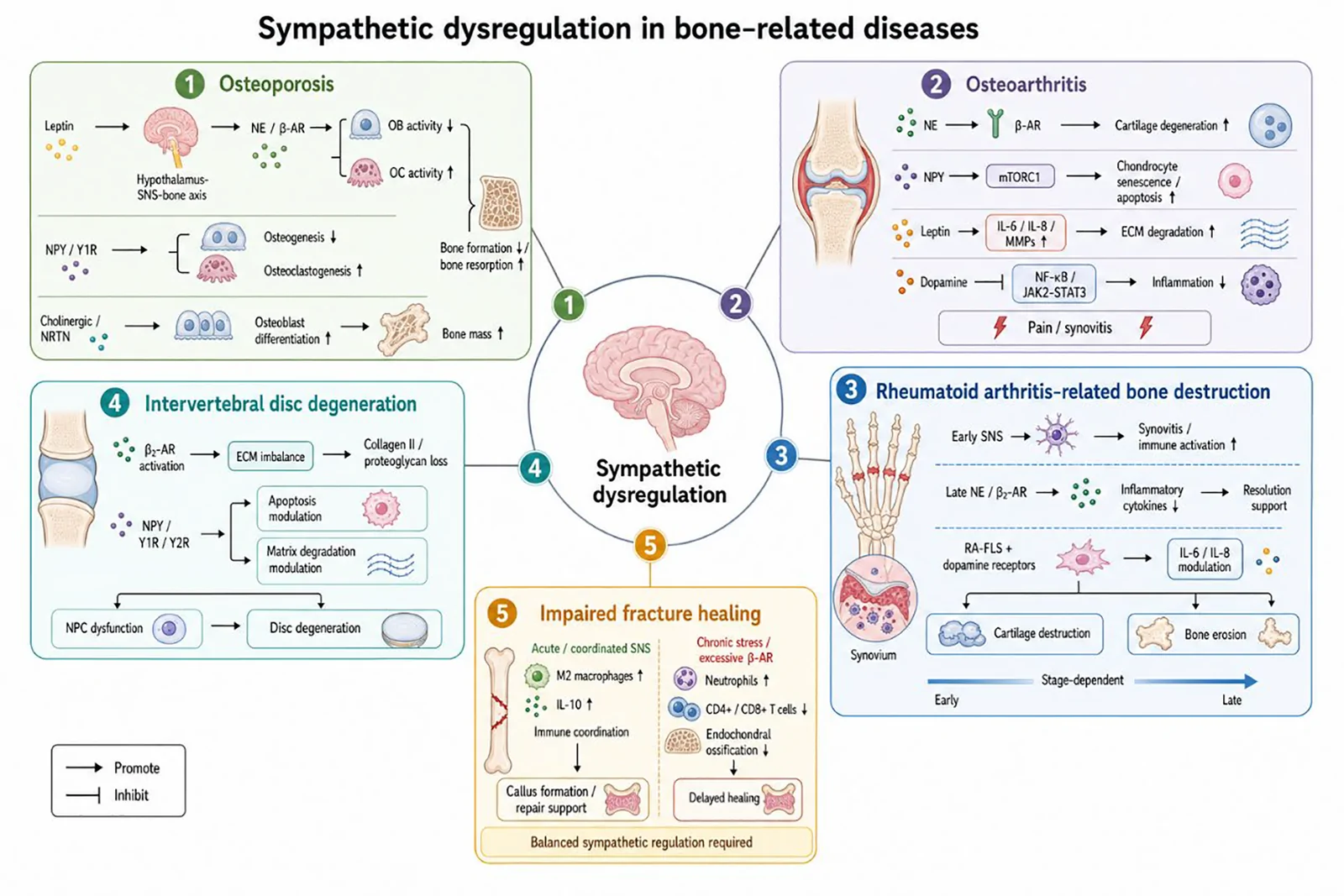
Sympathetic dysregulation in bone-related diseases. Sympathetic dysregulation contributes to multiple bone-related diseases through altered neuro-skeletal and osteoimmune signaling. (1) In osteoporosis, leptin–SNS–bone signaling, NE/β-AR activation, NPY/Y1R signaling, and cholinergic/NRTN-related pathways regulate osteoblast activity, osteoclastogenesis, bone formation, and bone resorption. (2) In osteoarthritis, sympathetic mediators, including NE, NPY, leptin, and dopamine, promote cartilage degeneration, chondrocyte senescence or apoptosis, extracellular matrix degradation, inflammation, pain, and synovitis. (3) In rheumatoid arthritis-related bone destruction, stage-dependent sympathetic signaling influences synovial inflammation, immune activation, inflammatory cytokine production, cartilage destruction, and bone erosion. (4) In intervertebral disc degeneration, β2-AR activation and NPY-related signaling contribute to extracellular matrix imbalance, collagen II/proteoglycan loss, nucleus pulposus cell dysfunction, apoptosis, and matrix degradation. (5) In fracture healing, coordinated sympathetic activation supports immune resolution and callus formation, whereas chronic stress or excessive β-AR signaling promotes inflammation, endochondral ossification, delayed callus maturation, and impaired repair.

The mechanisms discussed above indicate that sympathetic signaling regulates bone metabolism through both direct skeletal pathways and osteoimmune-mediated pathways. These mechanisms are not restricted to physiological remodeling, but are closely involved in several common bone-related diseases. In pathological states, sympathetic dysregulation may alter osteoblast activity, osteoclastogenesis, cartilage matrix metabolism, synovial inflammation, intervertebral disc homeostasis, immune-cell recruitment, and fracture repair (26, 30, 159). Therefore, sympathetic signaling provides a mechanistic link between systemic stress, neuroendocrine imbalance, local inflammation, and skeletal tissue degeneration. In this section, we discuss the role of sympathetic dysregulation in osteoporosis, osteoarthritis, rheumatoid arthritis-related bone destruction, intervertebral disc degeneration, and impaired fracture healing (**Figure 5**).

### Osteoporosis

Osteoporosis is characterized by reduced bone mineral density, deterioration of bone microarchitecture, increased bone fragility, and elevated fracture risk (204). Beyond the classical imbalance between osteoblast-mediated bone formation and osteoclast-mediated bone resorption, osteoporosis can also be interpreted in the context of neuroendocrine regulation of the skeleton. In this framework, hypothalamic outputs, sympathetic efferent activity, and local bone-cell responses converge to shift remodeling toward reduced formation and increased resorption. Sympathetic signals from the central nervous system can reach bone through peripheral pathways and regulate the skeletal microenvironment by promoting the release of norepinephrine (NE) and neuropeptide Y (NPY). NE mainly acts through β2-adrenergic receptors on osteoblast-lineage cells and osteocytes, suppressing osteogenesis and promoting osteoclastogenesis partly through RANKL release and miR-21-mediated signaling (205–208).

Excessive sympathetic activation is closely associated with osteoporotic bone loss. Inhibition of sympathetic activity promotes osteogenic differentiation of osteoblasts and mesenchymal stem/stromal cells, whereas sustained β-adrenergic stimulation impairs osteogenic differentiation, mineralization, and bone regeneration (206, 207). Similar β-adrenergic dominance, together with reduced α-adrenergic inhibitory tone, also aggravates bone loss in heart failure (205). Recent evidence has extended this mechanism to cancer treatment-associated skeletal injury. In tumor-bearing mice, localized radiotherapy induced systemic sympathetic activation and trabecular bone loss in distant vertebral, femoral, and tibial sites, accompanied by osteocyte apoptosis, enhanced osteoclast activity, and impaired osteoblast function; these abnormalities were attenuated by chemical sympathectomy (209). These findings indicate that persistent noradrenergic activation favors an osteoporotic remodeling pattern by inhibiting osteogenesis and stimulating osteoclast activity (209). Evidence from patients with catecholamine-secreting tumors provides clinical support for the skeletal consequences of sympathetic overactivation. Compared with patients with nonfunctioning adrenal tumors, patients with pheochromocytoma have been reported to exhibit increased bone resorption, lower lumbar trabecular bone quality, and impaired bone microarchitecture. The inverse association between urinary normetanephrine levels and trabecular bone score further suggests a relationship between chronic norepinephrine excess and skeletal deterioration. Nevertheless, these observational findings do not establish causality and may be influenced by endocrine and metabolic comorbidities (210). Recent evidence further indicates that sympathetic hyperactivation can impair vascularized osteogenesis by inducing metabolic dysfunction and senescence in type H endothelial cells. In preclinical Alzheimer’s disease mice, norepinephrine-mediated suppression of the c-Maf–PKM2 glycolytic pathway reduced endothelial pro-osteogenic support and contributed to early bone loss (211). However, sympathetic regulation is not uniformly catabolic, as sympathetic-associated cholinergic fibers and neurturin (NRTN) can promote BMSC osteogenic differentiation and improve bone microarchitecture in ovariectomized mice (26, 112–114).

NPY signaling further contributes to osteoporotic remodeling by inhibiting osteoblast proliferation and differentiation while promoting osteoclastogenesis (96, 97, 212). Genetic deletion of MSC-derived NPY or pharmacological blockade of NPY1 receptor enhances osteogenic marker expression, cortical bone strength, microdamage repair, and BMSC osteogenic differentiation through pathways such as cAMP/PKA/CREB (213, 214). In ovariectomy-induced osteoporosis, hypothalamic Y2 receptor antagonism reduces NPY release, increases bone density, and attenuates bone loss (99). Thus, central and peripheral NPY pathways may participate in osteoporosis depending on receptor subtype, cellular source, and disease context.

Leptin further illustrates the bidirectional nature of neuroendocrine regulation in osteoporosis. In osteoporosis, dysregulation of the central leptin–SNS axis may further enhance β2-adrenergic suppression of osteoblast-lineage activity. Norepinephrine released from sympathetic nerve terminals subsequently activates β2-adrenergic receptors on osteoblast-lineage cells, thereby suppressing bone formation (49, 142, 215). In aging and obesity-related metabolic disorders, leptin resistance, altered leptin receptor expression, and dysregulated NPY activity may further shift remodeling toward bone resorption (140, 142, 216, 217). Overall, osteoporosis may involve dysregulated neuroendocrine pathways that alter sympathetic activity and shift skeletal remodeling toward reduced osteoblast-mediated bone formation and increased osteoclast-mediated bone resorption (26, 30).

### Osteoarthritis

Osteoarthritis (OA) is a degenerative joint disease characterized by cartilage degradation, subchondral bone remodeling, osteophyte formation, synovial inflammation, pain, stiffness, and impaired joint mobility (218–221). Although traditionally regarded as a mechanical “wear-and-tear” disease, OA progression also involves neurogenic and sympathetic mechanisms (222–224). Sympathetic fibers are present in the synovium, periosteum, and subchondral bone, whereas normal adult articular cartilage is generally non-innervated. During OA progression, neurovascular invasion at the osteochondral junction and in osteophytes allows sympathetic-related mediators to enter the joint microenvironment, thereby influencing cartilage metabolism, subchondral bone remodeling, synovial inflammation, and pain responses (225, 226).

Norepinephrine (NE) regulates chondrocyte metabolism in a concentration- and receptor-dependent manner. The presence of adrenergic receptor subtypes and tyrosine hydroxylase in osteoarthritic chondrocytes indicates local adrenergic responsiveness. Low NE concentrations may attenuate IL-1β-induced matrix degradation and inflammation through β-adrenergic signaling, whereas high NE concentrations promote chondrocyte proliferation and apoptosis through α1-adrenergic signaling (227). NE also impairs chondrogenic differentiation, reduces aggrecan and type II collagen expression, and accelerates hypertrophic differentiation of progenitor cells (228, 229). Thus, abnormal adrenergic signaling may disturb chondrocyte survival, extracellular matrix homeostasis, and cartilage repair capacity. Consistent with this context-dependent pattern, Corrêa et al. found that surgical removal of the sympathetic supply to the temporomandibular joint exacerbated zymosan-induced mechanical hyperalgesia and selectively increased intra-articular IL-6, while TNF-α remained unchanged. Although this inflammatory pain model does not reproduce all features of chronic OA, it provides direct evidence that intact sympathetic innervation can exert local anti-hyperalgesic and cytokine-selective anti-inflammatory effects depending on the anatomical site, inflammatory stimulus, and measured endpoint (230).

Neuropeptide Y (NPY) further links sympathetic nerve infiltration to cartilage degeneration and pain-related dysfunction. During OA development, NPY-positive sympathetic fibers may enter cartilage through subchondral bone-associated vessels and release NPY into synovial fluid. Increased synovial fluid NPY levels are associated with middle- and late-stage OA and knee pain (231). Functionally, NPY aggravates cartilage matrix degradation, chondrocyte apoptosis, and inflammation by activating NPY2 receptor and mTORC1 signaling in articular chondrocytes, thereby promoting chondrocyte hypertrophy, matrix catabolism, and OA progression (232).

Leptin-related inflammatory signaling connects metabolic disorders with OA. In chondrocytes and synovial fibroblasts, leptin promotes IL-6, IL-8, nitric oxide, prostaglandins, and matrix metalloproteinase production through pathways including Ob-Rb/IRS-1/PI3K/Akt/AP-1 signaling, thereby amplifying inflammation and extracellular matrix degradation (233, 234). In obesity-associated OA, leptin also enhances chondrocyte–synovial fibroblast crosstalk and promotes chondrocyte senescence by inhibiting autophagy through mTOR activation (235–237). These findings suggest that OA progression is shaped by sympathetic mediators, metabolic inflammation, and cartilage matrix imbalance.

Notably, sympathetic-related signaling in OA is not exclusively detrimental. Dopamine can exert chondroprotective and anti-inflammatory effects by inhibiting NF-κB and JAK2/STAT3 signaling, and electroacupuncture may alleviate synovitis and referred pain by activating local sympathetic noradrenergic signaling (238, 239). Recent denervation evidence further supports a locally protective role of sympathetic innervation under specific inflammatory conditions. In a zymosan-induced temporomandibular joint inflammation model, superior cervical ganglionectomy aggravated mechanical hyperalgesia and selectively increased intra-articular IL-6 without altering TNF-α, indicating anatomically and cytokine-specific sympathetic regulation (230). Therefore, sympathetic modulation in OA should be interpreted in a context-dependent manner, with effects varying according to mediator type, receptor subtype, disease stage, and local joint microenvironment.

### Rheumatoid arthritis-related bone destruction

Rheumatoid arthritis (RA) is a chronic systemic autoimmune disease characterized by persistent synovitis, pannus formation, inflammatory cell infiltration, cartilage destruction, and periarticular bone erosion (240–242). Activated rheumatoid arthritis fibroblast-like synoviocytes (RA-FLS) are key effector cells in the destructive synovial microenvironment, where they display invasive behavior, produce inflammatory cytokines, interact with immune cells, and promote cartilage and bone destruction (243, 244). Because sympathetic fibers and catecholamine-related cells are present in synovial tissue, the SNS may influence RA-related bone destruction through neuroimmune and bone remodeling-related mechanisms.

Sympathetic signaling in RA is stage-dependent. In experimental arthritis, early sympathetic denervation ameliorates disease severity, whereas late denervation aggravates arthritis symptoms (245, 246). This discrepancy suggests that sympathetic activity may promote early inflammation but support inflammatory resolution at later stages. Denervation may also remove tyrosine hydroxylase-positive catecholaminergic cells with anti-inflammatory properties. In RA synovial tissue, loss of sympathetic nerve fibers is accompanied by increased norepinephrine (NE) release from synovial macrophages, indicating that local non-neuronal catecholamine production may compensate for sympathetic nerve loss (247). At relatively high concentrations, NE can activate β2-adrenergic receptors and suppress inflammatory cytokines such as TNF and IL-12 (220, 244). Thus, adrenergic signaling may either aggravate or alleviate RA inflammation depending on neurotransmitter concentration, receptor subtype, cellular target, and disease phase (245–247).

Dopaminergic signaling also participates in RA synovial pathology (248–250). Local dopamine synthesis occurs in RA synovial fibroblasts, macrophages, and B cells, and dopamine receptors are highly expressed in RA synovial fibroblasts (249, 250). Dopamine can inhibit IL-6 and IL-8 secretion, but it may also promote IL-6-dependent IL-17 production through D1-like receptors on CD4 naïve T cells; accordingly, D1-like receptor antagonism reduces cartilage destruction in RA models (248, 251). D2 receptor signaling has also been implicated in alleviating collagen-induced arthritis, and dopamine receptor modulation on osteoblasts may promote bone formation in arthritic microenvironments (252, 253). Thus, catecholamine-related signaling in RA depends strongly on cell type, receptor subtype, and pathological context.

Taken together, sympathetic signaling contributes to RA-related bone destruction through its interactions with immune and skeletal cells. Early excessive sympathetic activity may amplify synovitis and immune-cell activation, whereas later catecholamine-related signaling may support anti-inflammatory and pro-resolution responses. Therefore, the contribution of the SNS to RA should be understood as stage- and context-dependent, involving coordinated effects on synovial inflammation, immune-cell function, and local bone destruction.

### Intervertebral disc degeneration

Intervertebral disc degeneration (IVDD) is characterized by nucleus pulposus cell dysfunction, annulus fibrosus remodeling, extracellular matrix degradation, inflammatory activation, and progressive loss of disc structure and biomechanical function (254–258). Although healthy discs are largely aneural and avascular, degeneration is accompanied by neurovascular ingrowth, inflammatory mediator accumulation, and altered mechanical stress, providing an anatomical and microenvironmental basis for sympathetic-related mediators to participate in IVDD (257, 259–261).

Adrenergic signaling appears to be associated with IVDD severity (262, 263). Sympathetic nerve fibers and adrenergic receptors have been detected in healthy and degenerated disc tissues. During degeneration, α1-adrenergic receptor, α2-adrenergic receptor, and β2-adrenergic receptor expression are increased in vertebral endplate inflammation and structural alteration (262). In severely degenerated discs, enhanced and widely distributed β2-adrenergic receptor expression is positively correlated with IVDD pathological grade and extracellular matrix imbalance, including type II collagen degradation and proteoglycan loss (263). Thus, abnormal adrenergic stimulation may disturb disc matrix homeostasis and promote catabolic remodeling.

NPY-related signaling also changes during IVDD (264–266). Degenerative discs show increased expression of NPY and its receptors Y1R and Y2R compared with naturally aged discs (264). Under IL-1β-induced inflammation and abnormal mechanical stress, annulus fibrosus cells increase NPY synthesis and Y1R expression (265). However, NPY may exert protective effects in some degenerative contexts. In IL-1β-induced nucleus pulposus cell degeneration models, NPY reduces nucleus pulposus cell apoptosis by suppressing the mitochondrial apoptotic pathway and downregulates catabolic proteases such as MMPs, thereby delaying extracellular matrix destruction (266).

Taken together, sympathetic-related regulation in IVDD is context-dependent. β-adrenergic activation appears to promote extracellular matrix catabolism and disc degeneration, whereas NPY may attenuate inflammation-induced apoptosis and matrix degradation under certain conditions. Therefore, IVDD may involve an imbalance between catabolic adrenergic signaling and potentially protective neuropeptide responses.

### Impaired fracture healing

Fracture healing is a coordinated regenerative process involving hematoma formation, acute inflammation, soft callus formation, hard callus formation, and remodeling (159, 161). The sympathetic nervous system regulates osteoblasts, osteoclasts, vascular cells, immune cells, and skeletal progenitors during this process. Because fracture repair requires a transient inflammatory response that must be properly resolved, the effect of sympathetic signaling depends strongly on its timing, intensity, and duration (267–270).

Acute and coordinated sympathetic activation may support fracture repair by promoting an anti-inflammatory and pro-regenerative immune microenvironment. In traumatic brain injury combined with fracture, enhanced hypothalamic regulation of sympathetic activity increases M2 macrophage infiltration in callus tissue, expands anti-inflammatory myeloid cells, including Ly6_C_ monocytes and CD206^+^ macrophages, and elevates serum IL-10 levels (163). Sympathetic activation can also increase CD4^+^CD25^+^FoxP3^+^ regulatory T cells in bone marrow, suggesting that transient adrenergic signaling may facilitate immune resolution and regenerative coordination (169).

In contrast, chronic or excessive sympathetic activation impairs fracture repair. In chronic psychosocial stress and post-traumatic stress disorder models, increased neutrophil recruitment, reduced CD4^+^ and CD8^+^ T-cell populations, impaired endochondral ossification, and delayed callus formation can be partially rescued by propranolol, supporting the involvement of β-adrenergic signaling (164). Chronic stress-induced norepinephrine (NE) release also acts on β3-adrenergic receptors in bone marrow cells to suppress CXCL12 expression, thereby mobilizing neutrophils and inflammatory monocytes (165). In fracture settings, neutrophil-derived catecholamines may further activate adrenergic receptors on chondrocytes, forming a feed-forward inflammatory loop that disrupts callus formation and endochondral bone repair (166).

Importantly, complete loss of sympathetic input may also be detrimental. In ovariectomized fracture models, sympathectomy reduces CD8+ cytotoxic T cells and CD4+ helper T cells in fracture hematomas, which may impair callus maturation partly by reducing osteoprotegerin-producing CD4+ T-cell populations (173). Therefore, fracture healing requires balanced sympathetic regulation rather than simple sympathetic suppression. Physiological or acute activation may promote inflammation resolution, whereas chronic stress-related adrenergic activation may prolong inflammation and delay endochondral ossification. Therapeutic strategies should therefore aim to normalize sympathetic activity during specific repair phases rather than completely blocking sympathetic input. In osteoporotic fracture models, β-adrenergic blockade also enhanced the anabolic and regenerative effects of PTH, suggesting that sympathetic modulation may improve responsiveness to established bone-anabolic therapy (271). Despite these detailed experimental findings, direct evidence from human fracture cohorts remains scarce. It remains unclear whether the stage-specific changes in sympathetic activity, immune-cell recruitment, and adrenergic receptor signaling observed in animal models are reproduced during human fracture healing or predict delayed union and nonunion.

## Therapeutic strategies targeting sympathetic regulation in bone-related disorders

Therapeutic strategies targeting sympathetic regulation can be broadly classified into pharmacological modulation of sympathetic signaling and neuromodulatory interventions. Pharmacological approaches target β-adrenergic, NPY, and catecholaminergic signaling and may be delivered systemically or locally. Neuromodulatory approaches regulate sympathetic activity through physical or electrical stimulation.

### Pharmacological modulation of sympathetic signaling

#### β-adrenergic receptor blockade

β-Adrenergic receptor blockade represents the most extensively investigated pharmacological approach for modulating sympathetic effects on bone. Experimental evidence indicates that sustained β-AR activation suppresses osteoblast differentiation and promotes osteoclastogenesis, whereas propranolol can partially restore osteogenic activity under conditions of excessive sympathetic signaling. In osteoporotic fracture models, combined treatment with propranolol and parathyroid hormone produced greater anabolic effects than parathyroid hormone alone, suggesting that β-AR blockade may enhance the responsiveness of bone-forming cells to anabolic treatment (271). However, these findings remain predominantly preclinical and cannot be directly extrapolated to patients with osteoporosis or fractures.

Human observational evidence regarding β-blockers and skeletal outcomes remains inconsistent. In the Geelong Osteoporosis Study, β-blocker use was associated with higher BMD at the total hip and ultradistal forearm and with a lower risk of incident fracture (272). Similarly, the Dubbo Osteoporosis Epidemiology Study reported higher femoral-neck and lumbar-spine BMD and lower fracture risk among β-blocker users, with the association being more apparent for selective than for nonselective agents (273). A nationwide case–control study also found a small reduction in the risks of overall and hip fractures among β-blocker users (274).

Meta-analysis of 16 observational studies involving more than 1.6 million participants found an approximately 14% reduction in overall fracture risk among β-blocker users. The analysis also suggested that β1-selective blockers may be associated with greater fracture-risk reduction than nonselective agents (275). However, substantial heterogeneity was present across the included studies, and the apparent benefit was not consistently dose-dependent. In contrast, the Study of Osteoporotic Fractures found no association between β-blocker use and BMD and reported inconsistent associations with fracture risk (276). The SWAN cohort similarly showed that women initiating β-blockers had rates of BMD decline comparable to those of nonusers (277). These discrepancies may reflect differences in age, sex, cardiovascular indication, drug selectivity, exposure duration, fall risk, comorbidities, and concomitant medication use.

Randomized intervention studies provide a more cautious interpretation. In a placebo-controlled trial involving 41 postmenopausal women, propranolol at 160 mg/day for three months did not increase bone formation markers or BMD; instead, serum osteocalcin decreased, providing no evidence that nonselective β-blockade stimulates bone formation (278). A second 12-week randomized study in 32 postmenopausal women similarly found that propranolol did not significantly alter P1NP or CTX concentrations compared with the untreated control group (279).

By contrast, a later proof-of-concept trial involving 155 postmenopausal women reported receptor-selective effects. Twenty weeks of treatment with the β1-selective blockers atenolol and nebivolol reduced serum CTX and increased ultradistal-radius BMD, whereas propranolol did not produce comparable effects (71). These findings suggest that the skeletal effects of β-blockers in humans may depend more strongly on β-receptor selectivity than would be predicted from rodent studies, in which β2-AR signaling is generally considered dominant.

Human studies in osteoarthritis have also produced conflicting results. A large propensity score-matched primary-care cohort including 111,718 β-blocker users and an equal number of matched controls found that β-blocker prescription was associated with modestly lower cumulative risks of knee osteoarthritis and knee-pain consultations. Atenolol and propranolol were among the agents associated with lower consultation rates (280). In contrast, an eight-year longitudinal analysis of 1,168 participants with symptomatic knee osteoarthritis from the Osteoarthritis Initiative found no clinically meaningful association between β-blocker use and WOMAC knee pain, widespread joint pain, or analgesic use (281). Therefore, current observational evidence does not establish β-blockers as symptomatic or disease-modifying therapy for osteoarthritis, and randomized controlled trials would be required before clinical use could be considered.

Taken together, the available human evidence regarding the skeletal effects of β-blockers remains inconsistent. Observational studies suggest possible benefits for BMD, fracture risk, and musculoskeletal outcomes, whereas randomized studies, particularly those involving nonselective propranolol, have generally reported limited skeletal effects. Therefore, a cautious attitude should be maintained regarding the use of β-blockers in osteoporosis and osteoarthritis, and further studies are required to determine whether specific agents or patient populations may derive therapeutic benefit.

#### NPY receptor modulation

The NPY–Y receptor pathway provides another pharmacological target for sympathetic regulation of bone metabolism. Among the NPY receptor subtypes, Y1R and Y2R have received the greatest attention, but they appear to regulate bone through different anatomical and cellular mechanisms. Y1R is expressed mainly in peripheral skeletal cells, including osteoblast-lineage cells and osteocytes, whereas Y2R is more closely associated with central hypothalamic regulation. Therefore, receptor-selective targeting may allow different components of NPY-mediated skeletal regulation to be modulated.

Pharmacological inhibition of Y1R has shown anabolic and bone-protective effects in preclinical models. Oral administration of the selective Y1R antagonist BIBO3304 increased cancellous bone volume, trabecular number, trabecular thickness, and periosteal mineral apposition in mice, supporting the potential of peripheral Y1R blockade as an anabolic strategy (105). In human bone marrow stromal cells, genetic or pharmacological inhibition of Y1R enhanced osteogenic differentiation and RUNX2 expression through activation of the cAMP/PKA/CREB pathway (282). Y1R antagonism has also been investigated in ovariectomized rodent models, in which it improved bone microarchitecture and partly alleviated osteoporosis-associated changes by modifying gut microbial composition, intestinal barrier integrity, and circulating inflammatory exposure (174, 283).

Y2R antagonism may act through a more centrally mediated pathway. In ovariectomized mice, treatment with the brain-penetrant Y2R antagonist JNJ-31020028 increased whole-body BMD and improved vertebral and femoral trabecular microarchitecture while reducing circulating CTX-1 (99). Because direct exposure of isolated bone marrow cells to the antagonist did not alter osteoblast or osteoclast formation, these findings support a predominantly central mechanism. However, current evidence for both Y1R and Y2R targeting remains limited to cellular and animal studies. The systemic metabolic and neurological functions of NPY signaling, together with differences between central and peripheral receptor actions, will require careful evaluation before NPY receptor antagonists can be translated into clinical treatment for osteoporosis or other bone-related disorders.

#### Catecholaminergic modulation

Local catecholaminergic regulation may also be relevant in inflammatory joint disease. Human synovial-tissue studies have shown that rheumatoid arthritis is characterized by a marked loss of sympathetic nerve fibers together with increased catecholamine production by local immune cells, indicating a shift from neuronal to non-neuronal catecholaminergic regulation (247). Further studies using human rheumatoid arthritis and osteoarthritis synovial samples demonstrated the presence of tyrosine hydroxylase-positive catecholamine-producing cells in inflamed tissue. Pharmacological manipulation of intracellular catecholamine handling reduced inflammatory cytokine production in synovial-cell cultures and ameliorated experimental arthritis (246). Consistent with this concept, transplantation of tyrosine hydroxylase-positive catecholaminergic cells exerted anti-inflammatory effects in experimental arthritis (245). These findings suggest that targeted regulation of the local catecholaminergic environment may have therapeutic potential in inflammatory joint diseases. However, current evidence is derived mainly from human tissue analyses, ex vivo experiments, and animal models, and the optimal targets, intervention methods, and safety of catecholaminergic modulation require further investigation.

#### Localized delivery of sympathetic modulators

In addition to systemic administration, pharmacological modulation of sympathetic signaling may be achieved through localized delivery. Local delivery systems are designed to restrict drug exposure to skeletal lesions, enhance spatial control of sympathetic regulation, and potentially limit the cardiovascular, metabolic, and autonomic effects associated with systemic adrenergic intervention.

Local β-adrenergic blockade has been explored as a targeted strategy for bone regeneration. A three-dimensional collagen/polyvinyl alcohol/hydroxyapatite scaffold loaded with propranolol continuously inhibited local β-adrenergic signaling and promoted bone repair *in vivo*, whereas intraperitoneal propranolol did not produce a comparable regenerative effect (284). These findings suggest that sustained propranolol delivery at the defect site may alleviate local sympathetic inhibition of osteogenesis while limiting systemic β-adrenergic blockade.

Localized drug delivery may also be incorporated into multifunctional biomaterials. For example, the ZnS@PDA-PRO composite combines local propranolol release with mechanically responsive piezoelectric stimulation (285, 286). Because this system integrates localized pharmacological delivery with biomaterial-assisted neuromodulation, its neuromodulatory properties are discussed separately below.

Despite these potential advantages, localized delivery of sympathetic modulators remains predominantly preclinical. Local pharmacokinetics, drug retention, release kinetics, systemic exposure, long-term safety, scaffold degradation, and regenerative efficacy have not yet been adequately evaluated in humans.

### Neuromodulatory interventions

In addition to pharmacological modulation, sympathetic activity may be regulated through neuromodulatory interventions. These approaches use electrical, mechanical, or bioelectrical stimulation to influence local neural activity and nerve–tissue interactions, potentially providing greater spatial and temporal control than conventional systemic pharmacological treatment.

#### Electrical neuromodulation and electroacupuncture

Among neuromodulatory approaches, electroacupuncture has been investigated most extensively in osteoarthritis. In experimental osteoarthritis, electroacupuncture increased norepinephrine concentrations within the synovium without producing a corresponding systemic increase, thereby reducing inflammatory-mediator release, cartilage damage, and pain (239). This spatially restricted response suggests that, under specific local and experimental conditions, modulation of sympathetic signaling may exert anti-inflammatory effects without inducing widespread systemic adrenergic stimulation.

Human randomized trials have also evaluated electroacupuncture in patients with knee osteoarthritis. In a multicenter randomized sham-controlled trial involving 480 patients, intensive electroacupuncture administered three times weekly for eight weeks produced a higher combined pain and functional response rate than sham acupuncture, and the benefits persisted during follow-up (287, 288). Another randomized trial involving 301 patients showed that electroacupuncture improved pain, WOMAC scores, and conditioned pain modulation, with stronger stimulation producing greater improvements than weak or sham electroacupuncture (289). A recent systematic review and network meta-analysis further suggested that acupuncture, particularly electroacupuncture, may improve pain and physical function in knee osteoarthritis, although the certainty of evidence varied across comparisons (290, 291).

However, these clinical trials primarily evaluated symptomatic and functional outcomes and did not directly assess local sympathetic activity, norepinephrine release, or adrenergic receptor signaling. Therefore, although electroacupuncture has shown clinical benefits in knee osteoarthritis, whether these effects are specifically mediated by sympathetic neuromodulation remains to be established.

#### Biomaterial-assisted neuromodulation

Biomaterial-assisted neuromodulation represents an emerging strategy that combines physical stimulation with pharmacological regulation of sympathetic signaling. Piezoelectric biomaterials can convert mechanical loading into localized electrical signals, thereby providing mechanically responsive stimulation within the bone microenvironment.

A ZnS@PDA-PRO composite was developed to generate piezoelectric signals during mechanical loading while simultaneously releasing propranolol (285). The electrically active ZnS component provided local regenerative stimulation, whereas sustained propranolol release limited excessive β-adrenergic activation that could otherwise impair mesenchymal stem-cell osteogenesis. This combined strategy improved bone regeneration in critical-sized defect models.

This system therefore differs from conventional drug-delivery scaffolds because it integrates two potentially complementary mechanisms: local pharmacological blockade of β-adrenergic signaling and mechanically induced electrical neuromodulation. Such multifunctional biomaterials may provide spatially restricted and dynamically responsive regulation of nerve–bone interactions.

However, the relative contributions of piezoelectric stimulation, local propranolol release, and their combined effects on sympathetic signaling remain unclear. Future studies should separately evaluate these components and determine the optimal electrical output, drug-release profile, mechanical responsiveness, biodegradation characteristics, and long-term biosafety of these materials.

Taken together, current therapeutic approaches targeting sympathetic regulation can be broadly divided into pharmacological and neuromodulatory strategies. Pharmacological approaches include β-adrenergic receptor blockade, NPY receptor modulation, and catecholaminergic modulation and may be implemented through systemic administration or localized delivery. Neuromodulatory strategies include electroacupuncture and biomaterial-assisted electrical modulation. The effects of these interventions may vary according to molecular target, receptor subtype, anatomical site, disease context, treatment duration, delivery route, and intervention timing. Most localized delivery systems, catecholaminergic cell-based approaches, and biomaterial-assisted neuromodulatory strategies remain at the preclinical stage, whereas human evidence is currently concentrated mainly on β-blockers and electroacupuncture. Further mechanistic and clinical studies are needed to determine the appropriate therapeutic strategies for specific bone-related disorders.

## Conclusion

Bone metabolism is increasingly understood to be regulated by the nervous system in addition to the classical coupling between osteoblasts and osteoclasts. In this review, we identify the sympathetic nervous system as an important regulatory link connecting central neural activity, systemic stress responses, metabolic status, immune inflammation, circadian regulation, and local skeletal remodeling. This regulation involves adrenergic, NPY-related, and cholinergic/VIPergic signaling, together with upstream and local modulatory pathways such as the norepinephrine transporter, leptin, serotonin, endocannabinoid, and dopaminergic signaling.

By distinguishing direct skeletal regulation from indirect osteoimmune regulation, this review summarizes how sympathetic signaling affects bone formation, bone resorption, cartilage homeostasis, inflammatory remodeling, bone marrow niche function, and fracture repair. Importantly, these effects are context-dependent rather than uniformly anabolic or catabolic. Their skeletal consequences vary according to the anatomical site, target-cell population, receptor subtype, inflammatory condition, circadian phase, disease type, and stage of tissue injury or repair. This framework helps explain the different and sometimes opposing roles of sympathetic signaling in osteoporosis, osteoarthritis, rheumatoid arthritis-related bone destruction, intervertebral disc degeneration, and impaired fracture healing.

However, current evidence is still largely derived from animal models and *in vitro* studies, and its clinical relevance requires further validation. The spatiotemporal dynamics of sympathetic fibers, receptor expression, and circadian coupling remain incompletely defined. Further work is also needed to determine how neural signals interact with immune cells, blood vessels, and skeletal cells within local tissue niches. Future studies should clarify the timing, cellular targets, receptor specificity, disease-stage dependence, and therapeutic window of sympathetic regulation. Such evidence will be necessary to determine when sympathetic inhibition, receptor-selective modulation, neuropeptide targeting, local drug delivery, or neural intervention may provide clinically meaningful benefits in bone-related disorders.
